# Fast, accurate, point-of-care COVID-19 pandemic diagnosis enabled through advanced lab-on-chip optical biosensors: Opportunities and challenges

**DOI:** 10.1063/5.0022211

**Published:** 2021-09

**Authors:** Aref Asghari, Chao Wang, Kyoung Min Yoo, Ali Rostamian, Xiaochuan Xu, Jong-Dug Shin, Hamed Dalir, Ray T. Chen

**Affiliations:** 1 Department of Electrical and Computer Engineering, The University of Texas at Austin, Austin, Texas 78758, USA; 2 Omega Optics, Inc., 8500 Shoal Creek Blvd., Austin, Texas 78757, USA

## Abstract

The sudden rise of the worldwide severe acute respiratory syndrome coronavirus 2 (SARS-CoV-2) pandemic in early 2020 has called into drastic action measures to perform instant detection and reduce the rate of spread. Common clinical and nonclinical diagnostic testing methods have been partially effective in satisfying the increasing demand for fast detection point-of-care (POC) methods to slow down further spread. However, accurate point-of-risk diagnosis of this emerging viral infection is paramount as the need for simultaneous standard operating procedures and symptom management of SARS-CoV-2 will be the norm for years to come. A sensitive, cost-effective biosensor with mass production capability is crucial until a universal vaccination becomes available. Optical biosensors can provide a noninvasive, extremely sensitive rapid detection platform with sensitivity down to ∼67 fg/ml (1 fM) concentration in a few minutes. These biosensors can be manufactured on a mass scale (millions) to detect the COVID-19 viral load in nasal, saliva, urine, and serological samples, even if the infected person is asymptotic. Methods investigated here are the most advanced available platforms for biosensing optical devices that have resulted from the integration of state-of-the-art designs and materials. These approaches include, but are not limited to, integrated optical devices, plasmonic resonance, and emerging nanomaterial biosensors. The lab-on-chip platforms examined here are suitable not only for SARS-CoV-2 spike protein detection but also for other contagious virions such as influenza and Middle East respiratory syndrome (MERS).

## I. INTRODUCTION

### A. Novel respiratory virus infection and importance of highly sensitive point-of-care detection

The emergence of novel respiratory tract infections in the 21st century has been a growing concern that has turned into a major cause of concern, hospitalization, and death. The growing alert turned into a full-scale, worldwide deadly pandemic in 2020 with SARS-CoV-2 (severe acute respiratory syndrome coronavirus 2) following predecessor respiratory pandemics, including SARS (severe acute respiratory syndrome) in 2003, H1N1 influenza (swine flu) in 2009, and MERS (Middle East respiratory syndrome) in 2012. The coronavirus disease 2019 (COVID-19) pandemic and its rapid growth rate have driven an unprecedented worldwide demand for emergency measures to mitigate its fast rate of spread.^1–3^ In addition to inevitable policies like social distancing, laborious sterilization measures, and protocols for coping with infected patients, it is paramount to have a system of detection and cure in place for a catastrophic pandemic of which very few humans alive have experienced. It is also well known that pressures of war have always stimulated advances in engineering, science, and medicine. Therefore, the new, invisible battle against SARS-CoV-2 infection can stimulate major breakthroughs in the development of diagnosis and treatment systems. The highly contagious SARS-CoV-2 infection is hard to detect, as patients present with clinically inapparent symptoms, including fever, cough, and shortness of breath.^4^ The worldwide high morbidity and mortality of SARS-CoV-2 plus no guaranteed vaccine or treatments on the horizon as of mid- to late-2020 are a call to action for scientists and researchers to probe various medical interventions. Immediate, cost-effective, point-of-risk measures, including identification, diagnosis, and isolation of the infected individual, are still regarded as the single best viable solution to slow down this epidemic pneumonia.

### B. Structure of SARS-CoV-2

**FIG. 1.**
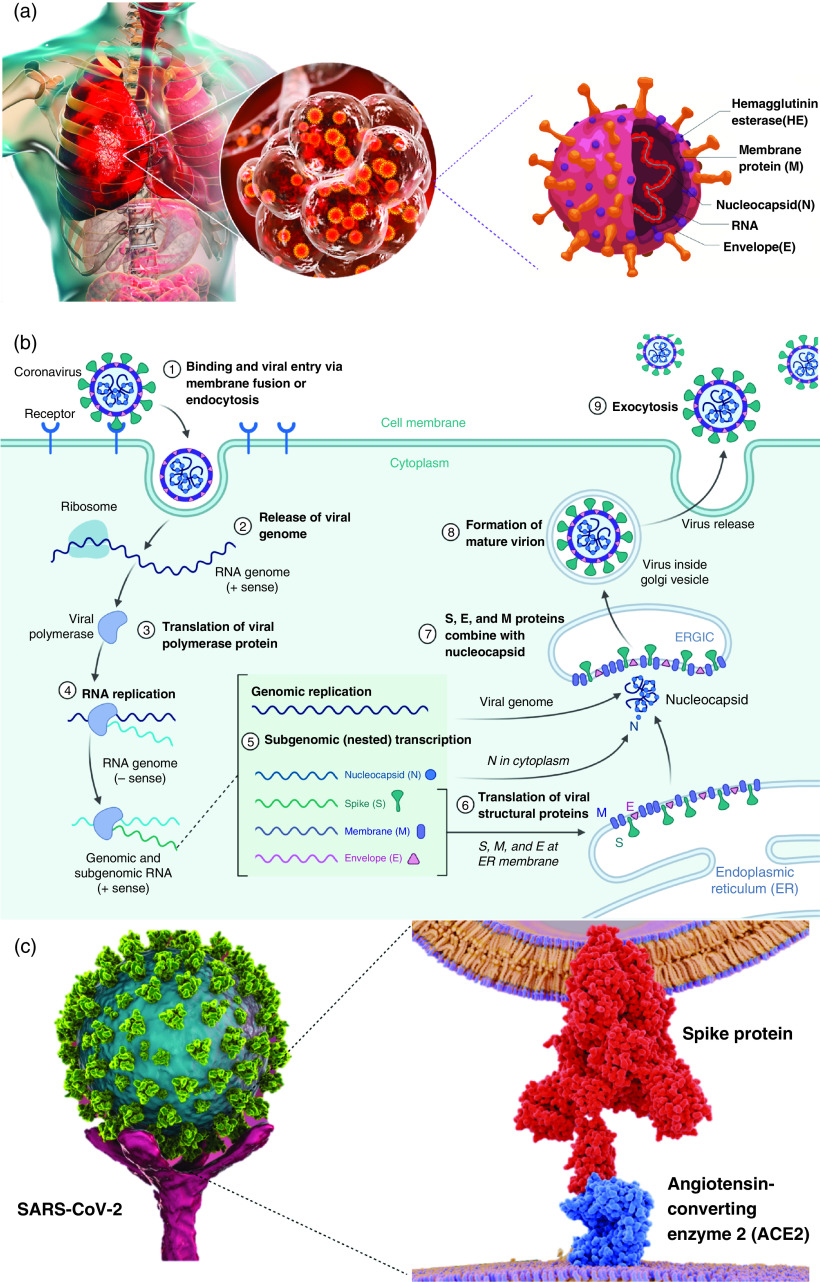
(a) Colorized schematic illustration depicting a heavily infected lung by SARS-CoV-2 virus molecules. SARS-CoV-2 molecular structure is illustrated in detail as well. The RNA and membrane protein are significant as they provide great affinity to bioreceptors functionalized on the surface of the biosensor. (b) Life cycle of pathogenic human SARS-CoV. The virus enters the target cell through respective cellular receptor angiotensin-converting enzyme 2 (ACE2) on the membranes of host cells. Viral genomic RNA is then unveiled in the cytoplasm and translated into viral polymerase proteins. Viral RNA and nucleocapsid (N) structural protein are replicated and transcribed in the cytoplasm to form a mature virion and then released from host cells.^12^ Adapted with permission from Jiang *et al.*, Trends Immunol. **41**, 355 (2020). Copyright 2020 Elsevier.^12^ Panel (b) was created using BioRender (https://biorender.com/). (c) The virus enters the target cell by first binding its S glycoproteins to the respective cellular ACE2 on the membranes of host cells, which mediate virus–cell membrane fusion and viral entry (left). Schematic of SARS-CoV-2 virus binding to ACE2 receptors on a human cell (right). Schematic shows that the coronavirus spike protein (red) mediates the virus entry into host cells. It binds to ACE2 (blue) and fuses viral and host membranes.

SARS-CoV is an enveloped, single-stranded RNA virus that exists in humans and animals and is mainly transmitted through aerosols and nearby interpersonal contact.^5,6^ RNA viruses like SARS-CoV-2 usually have the length of 2–32 kb, and SARS-CoV-2 possesses the largest genome size of any known RNA virus with a length of 30 kb and an S protein trimer of 600 kDa. Once the virus enters the body, it sticks to primary target cells that provide plenty of virus receptors, the angiotensin-converting enzyme 2 (ACE2).^5,7^ Its genome RNA infusion into the cell results in the formation of protein building blocks consisting of spike, envelope, membrane, nucleocapsid, and proteins.^8–10^ Thus, relying on ACE2, human SARS-CoV infuses into the target cell for which S glycoprotein trimeric spikes on the surface mediate the entrance into the host cell.^11^ The S glycoprotein of SARS-CoV is therefore a main target for neutralizing antibodies.^12^ Similar SARS-CoV and SARS-CoV-2 amino acid identity in their S proteins makes them prone to have analogous immunogenic surfaces on these antigens.^13^ SARS-CoV-2 demonstrates a complex pattern for receptor recognition that results in its trimeric spike protein attachment to ACE2 receptors on human cells^7,14–18^ [Fig. 1(c)]. Attempts to block the infusion of virus have been carried out through targeting mainly the spike protein of SARS-CoV-2 and the receptor binding domain (RBD).^19^ Developed antibodies targeting these regions can expand the potency, power, and chance of success against the infusion of SARS-CoV-2 in the host cell. Once the virus enters the body through the cells, it replicates, and virions are then set free to infect new target cells^16,20^ [Fig. 1(b)]. SARS infectious virion particles can be easily found in respiratory secretions, saliva, and sweat within the early days of effective infection.^21–26^ SARS-CoV-2 infection harms lung tissues, resulting in pneumonia with rapid respiratory deterioration, failure, and death in almost 5% of cases.^27,28^ Applying an effective vaccine against SARS-CoV-2 can be done through the S protein and especially the RBD as they induce a highly potent neutralizing antibody to block virus binding and its membrane infusion or form an immunity protective layer against viral infection.^5^

### C. Detection mechanisms for SARS-CoV-2 viral infection

The most well-known and commonly used procedure for identifying pathogens like SARS-CoV-2 relies on clinical real-time RT-PCR (reverse transcription polymerase chain reaction).^29^ The high accuracy and precision of the RT-PCR clinical test at early stages of infection in a symptomatic patient makes it the most reliable method to detect SARS-CoV-2 to date. The test is specifically done for a qualitative analysis of nucleic acid of the pathogen in people who meet SARS-CoV-2 clinical infection signs and symptoms. For infected patients who are asymptomatic or identified as false negative with a viral load below the detection limit of RT-PCR assays, serological tests are the second-best alternative and can be used to further control the virus spread. However, the aforementioned procedures and similar clinical diagnosis all require advanced laboratory testing, expensive equipment, and expertise, which are counterproductive for the purpose of point-of-care (POC) fast detection and hard to be found in low- and middle-income countries (LMICs) that are more prone to suffer from the outbreak.^22,29–32^ The increasing number of potentially positive people and the inability to test on a mass scale due to the high-cost and time-consuming nature of clinical diagnosis procedures have imposed the need to develop methods that are more conducive to fast, cost-efficient detection of SARS-CoV-2.

**FIG. 2.**
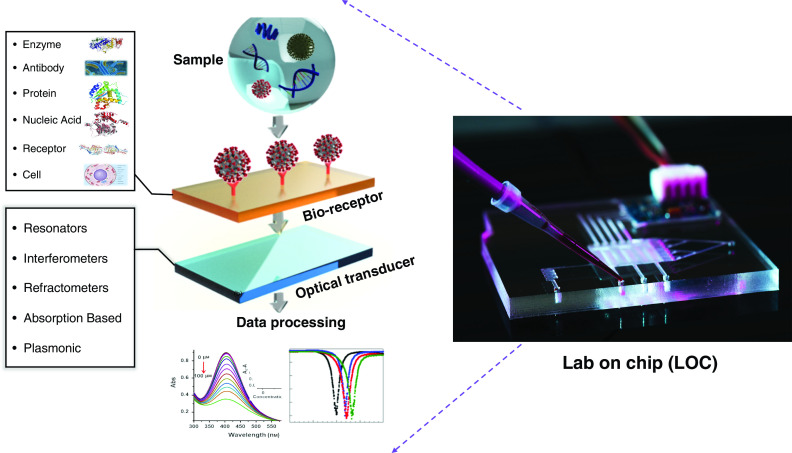
Schematic working principle of a lab-on-chip (LOC) optical biosensor. Abs, absorbance.

POC biosensors can provide the next best alternative reliable solution to clinical diagnosis with a much faster real-time detection without compromising sensitivity and accuracy.^33,34^ The World Health Organization has defined and specified the standards and requirements of a convenient biosensor to be used throughout the world as ASSURED: affordable, sensitive, specific, user-friendly, rapid and robust, equipment free, and deliverable to end-users.^35,36^ Compatible with these requirements, there have been many POC nucleic acid biosensor platforms introduced, developed, or modified to detect SARS-CoV-2 by ASSURED standards.^35,37,38^ However, a major challenge for all available biosensors is early confirmation of SARS-CoV-2 with a low level of viral RNA at the onset of the infection to avoid further spread.^39,40^ Among all the portable platform solutions to this task, optical biosensors (Fig. 2) present a strong conceivable potential to grow expeditiously in healthcare and biomedical fields as they provide a condensed, accurate analytical tool to promote mass-scale screening of a broad range of samples through different parameters.^40–43^ The main advantages to optical biosensors are delivering the accuracy of nucleic acid–based clinical tests while eliminating the need to postprocess the extracted sample or collected serum.^44^ As a result, they are very promising to become the future COVID-19 diagnostic tools as they provide sensitive and durable point-of-care testing (POCT) devices, which is imperative for controlling epidemics like SARS-CoV-2.^39,42,45–48^

Optical biosensing works under different physical transduction principles, such as interferometers, resonators, and plasmonics,^49^ and has been investigated in several studies to monitor many viruses with a good accuracy.^24,45,50^ By exploiting the strong light–matter interactions in a POC optical biosensor, an ultrasensitive real-time detection platform for different pathogens, including novel SARS-CoV-2, is achievable. An optical biosensor essentially translates the capture of the virion in a measurable alteration of a light property, such as the refractive index (RI), intensity, or resonance shift, through different methods such as resonators and interferometers (Fig. 2).

### D. Label-based vs label-free detection mechanisms

**FIG. 3.**
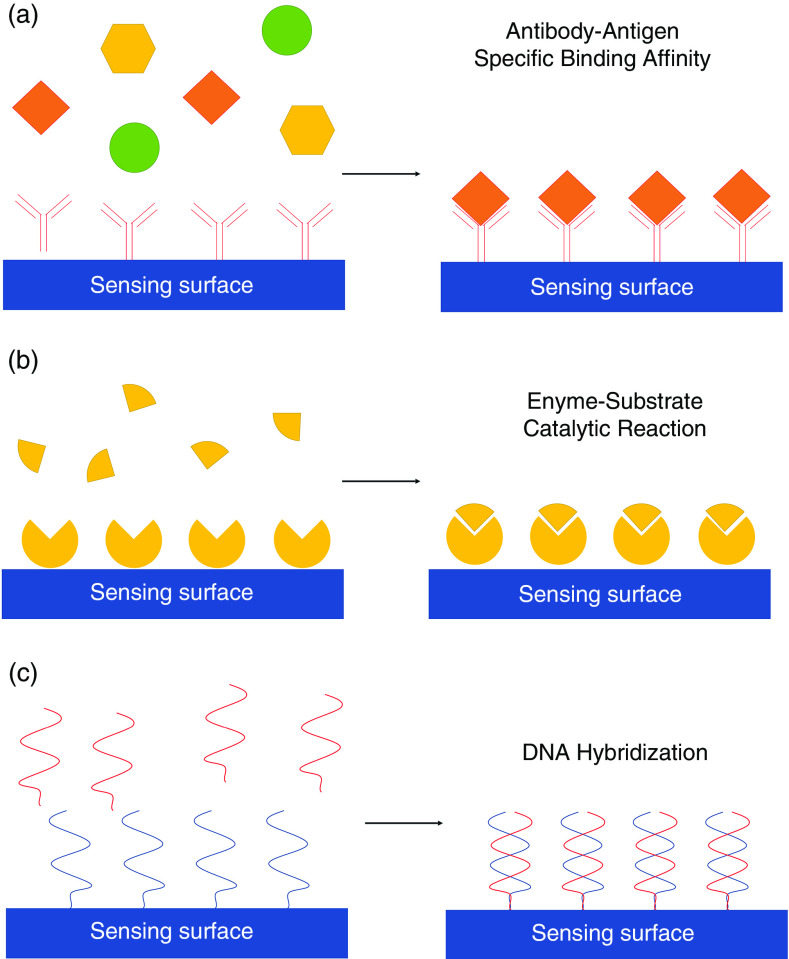
(a)–(c) Various receptor–target coupling mechanisms on the biosensor surface showing antibody–antigen binding (a), enzyme–surface catalytic reaction (b), and DNA hybridization (c). (d) Schematic of SARS-CoV-2 virus binding to ACE2 receptors on a human cell. Inset shows that the coronavirus spike protein (red) mediates the virus entry into host cells. It binds to the angiotensin converting enzyme 2 (blue) and fuses viral and host membranes.

A biosensor functioning mechanism, in general, consists of a sample or target analyte that binds to the bioreceptor, after which the transducer helps in converting the biorecognition data into a measurable quantity (Fig. 2). The difficulty in directly detecting biological analytes based on their physical properties has led to labeling techniques in which an additional molecule is attached to immobilized target molecules, viruses, or cells to enhance the quantitative signal.^41^ Therefore, the specific immobilized bioreceptors on the chip sensing area will be bound to their targeted labeled pathogens or proteins upon introduction of analytes into the sensing area. Notable examples of labels used in biosensing are dye molecules, fluorescent tags, and enzymes. Various types of bioreceptor–target coupling mechanisms have been demonstrated (Fig. 3), including antibody–antigen binding, enzyme–substrate catalytic reaction, and complementary DNA (cDNA)–DNA hybridization.

These labels require sophisticated reagent selection and modification that in turn can come with the drawback of perturbing the assay and, in some cases, making final detection a challenging task. On top of that, labeling chemistry can be expensive and time consuming. Thus, there have been many intriguing efforts in biosensing systems involving unlabeled or unmodified biomolecules (label-free biosensing)^41,42,51,52^ in which native molecular properties, like molecular weight and RI, are utilized for sensing. Label-free detection, on the other hand, has its own shortcomings as well; for instance, it requires low, nonspecific binding^49^ and sufficient signal to be generated upon target binding.^42^ However, its benefits, such as providing real-time analysis by simplifying assays and reducing time and number of steps required as well as eliminating experimental uncertainty, can compensate its limitations provided that the target analyte concentration and its surface adsorption are sufficient enough to enable detection.^53^

In the case of an optical biosensing platform, the sensing transduction signals are usually based on miniscule changes in refractive index, which itself results from the attachment of biomolecules to the immobilized bioreceptors. The biosensor final sensitivity and specificity are also strongly dependent on the immobilized molecules and the accessibility of target analytes to them. In a label-free biosensor, a highly sensitive biorecognition layer on the transducer surface is therefore of vital importance.^49,54,55^ Hence, the optimization of sensing surfaces and their biofunctionalization strategies becomes a significant element for any accurate, sensitive, label-free biosensor.^49^ However, the diverse range of target molecules and biosensor applications makes obtaining a universal surface biofunctionalization procedure extremely difficult; consequently, the procedure needs to be custom designed. For instance, in graphene-based field effect biosensing (FEB), the surface is functionalized for protein immobilization with anti-Zika NS1 mouse monoclonal antibody (mAb) 6B1 developed by the Centers for Disease Control and Prevention.^56^ Polyethylene glycol (PEG) has also been studied for removing proteins from the surface of silicon-based biosensors as it provides a stable and anti-absorptive block against undesired, nonspecific interactions.^57,58^ Putting the difficulty of abundant surface functionalization methods aside, the main challenge for label-free biosensing compared to label-based biosensing is achieving the desired sensitivity and limit of detection (LOD) without increasing the target analyte concentration. For detecting pathogens like SARS-CoV-2, a variety of factors can determine the effectiveness of labeled vs nonlabeled procedures.

All in all, for a highly sensitive optical biosensor suitable for detecting SARS-CoV-2, labeled or label free, the limit of detection is the determining factor to have a reliable POC substituting the lab-based detection methods. In Secs. II–V, we discuss the most sensitive optical biosensors that can be used as a platform for a highly sensitive fast point-of-care detection system along with the latest advancement as well as associated challenges.

## II. INTEGRATED OPTICAL BIOSENSORS

**FIG. 4.**
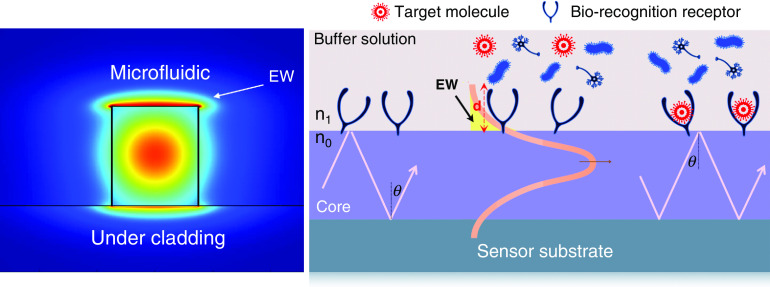
(a) Schematic of a waveguide-based biosensor facilitated through a biorecognition element attached on its surface to bind with the COVID-19 molecule. EW, Electromagnetic Wave.

Due to their immunity to electromagnetic interference (EMI), compactness, and high selectivity, optical biosensors have attracted special attention as a biosensing system.^59,60^ The interaction of target molecules with bioreceptors on the surface leads to an effective index and absorption coefficient change (Fig. 4). The effective index change is a function of the concentration of biological or chemical targets on the surface.

Effective mode index change for a perturbed waveguide can be calculated through the variation method:^52^

$$\Delta n_{\mathrm{eff}}=\frac{{n_{m}^{2}-n}_{c}^{2}}{Z_{0}P}\iint {\left|E(x,y)\right|}^{2}d\mathrm{xdy},$$ (1)

where E(x,y) is the electric field $;\;Z_{0}$ is the free space impedance; P represents the light wave power, and $n_{c}\;\mathrm{and}\;n_{m}$ are the refractive indexes of the aqueous solution without analyte and molecular adsorption layer, respectively.

Sensitivity (S) and LOD are the two main criteria for evaluating the performance of the optical biosensor, which in turn depends on the strength of the interaction between the substance and light in a solution or on the surface.^60^ Here, we further investigate the most well-defined biophotonic-sensing mechanisms based on interferometer and resonance shift in microcavities.

### A. Integrated interferometer sensing

**FIG. 5.**
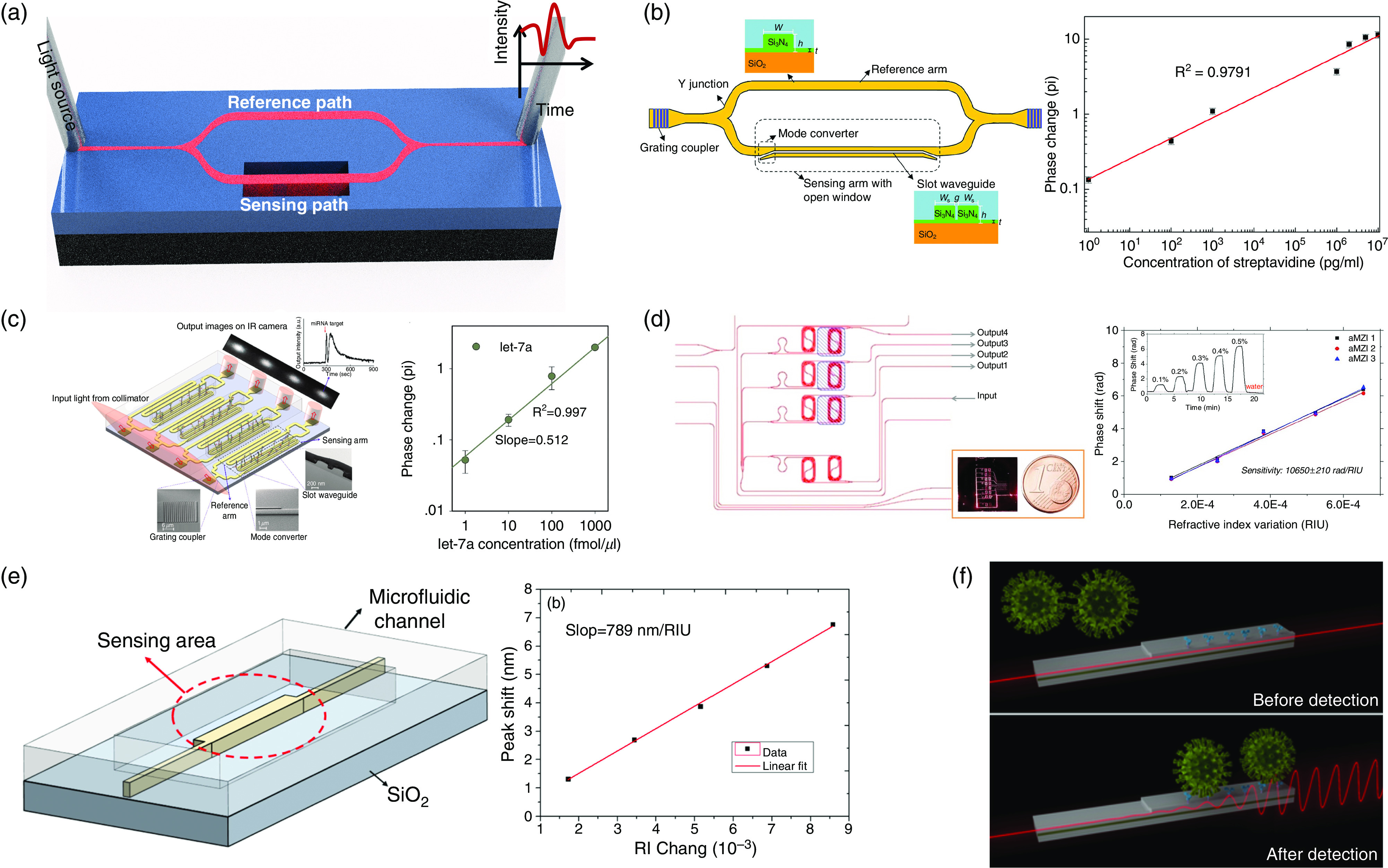
(a) Schematic diagram of MZI biosensor.^63^ The MZI biosensor includes a reference arm and a sensing arm connected to two Y junctions. (b) Left: An MZI biosensor with a silicon nitride strip waveguide as the reference arm and a silicon nitride slot waveguide as the sensing arm. Right: the phase change induced by streptavidin binding as a function of the concentration with the range of 19 fM to 190 nM,^63^ which provides a linearity of R^2^ = 0.9791. (c) Left: Schematic diagram of silicon nitride based on MZI used to detect miRNA in human urine samples. Right: Phase change to the binding of target miRNAs with a linear fitting curve of R^2^ = 0.997.^25^ Reprinted with permission from Liu *et al.*, Biosens. Bioelectron. **71**, 365 (2015). Copyright 2015 Elsevier.^25^ (d) Left: Schematics of an asymmetric MZI biosensor with purified solutions. Right: Volume sensitivity measurements with ∼10 650 rad/RIU.^66^ Inset: Phase shift curve for an MZI biosensor with different concentrations of solutions. (e) Schematic structure of the polymer-based bimodal interferometer.^67^ A 5-mm sensing length provides sensitivity of 316*π* rad/RIU and an extinction ratio of 18 dB. (f) A novel laser-based BMW (bimodal waveguide) sensor proposed to detect the COVID-19 via alteration in the evanescent light field.

Integrated interferometer photonics is one of the most practical architectures for sensing applications. It is based on splitting the input beam into two arms through a Y junction: One arm is completely retained as the reference arm, and the other arm contains the target. The interaction of electromagnetic waves on the sensing arm will cause a phase difference with respect to the reference arm, and recombination of the two beams in the output will cause constructive or destructive interference [see Fig. 5(a)].

Output intensity $I_{\mathrm{Out}}$ of the Mach–Zehnder interferometer (MZI) is described as follows:^48,61,62^

$$I_{\mathrm{Out}}=I_{\mathrm{sen}}+I_{\mathrm{ref}}+2\sqrt{I_{\mathrm{sen}}I_{\mathrm{ref}}}\text{ cos}\left(\varphi_{0}+\Delta \varphi \right),$$ (2)

where $I_{\mathrm{ref}}$ and $I_{\mathrm{sen}}$ are light intensity in reference and sensing arms, and $\varphi_{0}$ is the initial phase difference between two arms without external perturbation. The sensitivity of the MZI-based sensor is related to the phase sensitivity relative to the length of the sensor arm,

$$S_{ph}=\frac{\Delta \varphi }{{\Delta n}_{\mathrm{eff}}L}.$$ (3)

In an imbalanced MZI, considering the phase matching condition, vis-à-vis the wavelength sensitivity, we can approximate the phase sensitivity of the MZI-based sensor,

$$S_{ph}=\frac{2\pi }{\Delta \lambda }\frac{S_{\mathrm{FSR}}}{L},$$ (4)

where Δλ is the free spectral range (FSR) and $S_{\mathrm{FSR}}$ is the spectral sensitivity.

Chemical and biosensing via MZIs have been widely exploited,^63^ and judicious design of imbalanced interferometers based on waveguides and photonic crystal waveguides (PCWs) have been explored by a lot of research groups.^42,64,65^ Figure 5(b) shows an MZI biosensor with a silicon nitride strip waveguide as the reference arm and a silicon nitride slot waveguide as the sensing arm. Sensitivity of as low as 18.9 fg/ml (fM) and bulk refractive index sensitivity of 1864 *π*/refractive index units (RIU) are obtained through increasing light–analyte interaction in the slot waveguide-sensing region thanks to the low refractive index subwavelength-sized slot regions. Liu *et al.*^25^ designed a slot waveguide in the sensing arm to maximize the overlap between light and target analyte [see Fig. 5(c)]. They reported a wide range of linear tests with concentrations ranging from 19 fM to 190 nM (R^2^ = 0.979177). The asymmetry of the MZI array is used to detect microRNA (miRNA) in human urine samples by measurement of light phase change due to attachment of complementary DNA capture probe and the target miRNA. Utilizing slot waveguide as the sensing arm plus the elimination of the tedious incubation process make it an integrated, accurate, and sensitive POC optical biosensor. Chalyan *et al.*^66^ demonstrated detection of aflatoxin M1 through asymmetric Mach–Zehnder interferometer surface functionalized with antibody fragments. They demonstrated a high volumetric sensitivity of 10^4^ rad/RIU, leading to a LOD <5 × 10^−7^ RIU enabled through surface functionalization of the biosensor [Fig. 5(d)].

As is shown in Fig. 5(e), Zhao *et al.*^67^ demonstrated a polymer-based lateral bimodal interferometer. They used two transverse modes in the waveguide to create the interferometer and short wavelength of 890 nm to reduce the absorption loss from the aqueous solution to be sensed. The overall sensitivity of the manufactured interferometer sensor with a sensing length of 5 mm is reported to be 316*π* rad/RIU, and the extinction ratio can reach 18 dB. However, reliability of the polymer needs to be further studied. An analogous platform with a laser-based bimodal waveguide interferometer is also proposed to detect COVID-19 via changes in the sensor's evanescent light field [Fig. 5(f)]. This nanophotonic POC device can directly examine body respiratory fluids (e.g., saliva) and does show a sensitivity of attomolar (aM) level for direct, specific miRNA targeting.

### B. Resonance shift sensing

In contrast to waveguide-based sensors that rely on light wave absorption, resonant displacement in functionalized microcavities provides a wide range of ultrasensitive optical biosensors.^68,69^ The magnitude of binding is determined by de Feijter's formula,^70^ which relates the absolute quantity of adsorbed molecules *M* with the change in the refractive index as

M=dAnA−ncdndt, (5)

where *d_A_* is the thickness of the adsorbed layer, *n_A_* is the refractive index of adsorbed molecules, *n_c_* is the refractive index of the cover solution, and *dn*/*dt* is the change in the refractive index of molecules, which is proportional to the shift *dλ* in position of the resonance peak. The size of the resonance wavelength shift is proportional to the number of adsorbed biomolecules, thus providing a label-free method to quantitatively determine the target analyte.

#### 1. Ring resonators

**FIG. 6.**
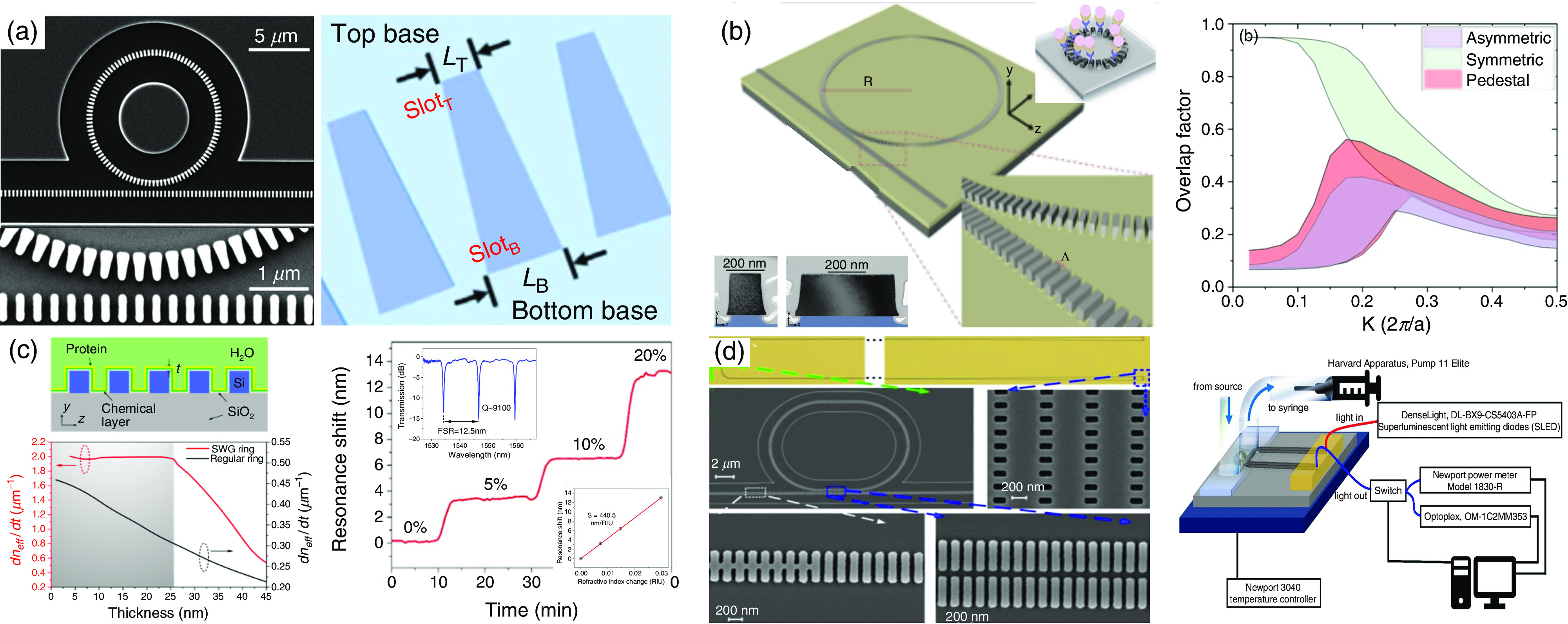
Subwavelength grating waveguide-based micro-ring resonator (SWGMR)/racetrack biosensors. (a) High-Q SWGMR based on trapezoidal pillars.^71,72^ Reprinted with permission from Wang *et al.*, Opt. Lett. **41**, 3375 (2016). Copyright 2016 The Optical Society,^71^ and Wang *et al.*, Sci. Rep. **6**, 24106 (2016). Licensed under a Creative Commons Attribution (CC BY) license. (b) Pedestal SWGMR-based biosensor for improved S.^74^ Reprinted with permission from Chang *et al.*, Biosens. Bioelectron. **141**, 111396 (2019). Copyright 2019 Elsevier.^74^ (c) T-SWGMR–based high-Q and high-S biosensor.^73^ Reprinted with permission from Hai *et al.*, Opt. Express **24**, 29724 (2016). Licensed under a Creative Commons Attribution (CC BY) license.^73^ (d) Racetrack SWGMR biosensor with high Q and high S.^75^ Reprinted with permission from Huang *et al.*, Opt. Express. **25**, 10527 (2017). Licensed under a Creative Commons Attribution (CC BY) license.^75^

Although high-quality (Q) ring resonators can be achieved with a larger radius, the trade-off between the Q and the FSR limits the radius for a given FSR, which should be large enough for effective recognition of the sensing signal from the adjacent interference signals or for large-scale on-chip multiplexing sensing applications. Wang *et al.*^71,72^ proved through experiments that the Q-enhanced subwavelength grating waveguide-based metamaterial ring resonator (SWGMR) was, in particular, designed using a trapezoidal silicon column (T-SWGMR) (Fig. 6). Contrasted with conventional rectangular silicon pillars comprising SWGMRs (R-SWGMRs), an asymmetric effective refractive index distribution is created, which can significantly reduce bending loss and thus increase the Q of SWGMRs.

The experimental results show that the applicable Q value of T-SWGMR with a radius of 5 *μ*m is as high as 11 500, which is 4.6 times the Q value (∼2800) provided by R-SWGMR with the same radius, indicating that the propagation loss is reduced by 81.4%. To go one step farther, Hai *et al.*^73^ proposed a T-SWGMR biosensor and demonstrated the unique stable surface sensing characteristics through a demonstration of miRNA detection at a concentration of 1 nm [Fig. 6(b)].

In addition to utilizing the unique stable sensing characteristics of SWGMR and the enhanced Q of T-SWGMR, Chang *et al.*^74^ showed a pedestal T-SWGMR biosensor that maximizes the mode volume overlap by implementing an asymmetric refractive index distribution along the vertical direction on the silicon-on-insulator (SOI) platform, thereby further improving sensitivity [Fig. 6(d)]. Both theoretic analysis and experimental proofs show that the volume sensitivity and surface sensitivity have been significantly increased by 28.8% and 1000 times, respectively. For streptavidin, a spectrometer with a resolution of 0.01 nm is used, and its LOD is ∼400 fM. Owing to an imperfect manufacturing process, experimental Q estimate of T-SWGMR with a radius of 10 *μ*m and FSR of ∼13 nm is 1800. The optimized SWGMR with symmetric coupling demonstrated by Huang *et al.*^75^ estimated Q to be 9800.

#### 2. Microtoroid

**FIG. 7.**
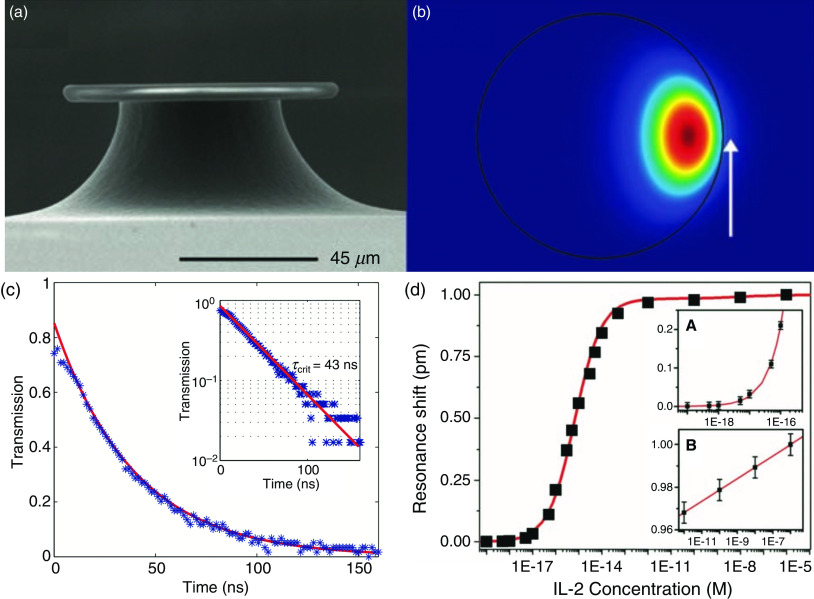
The cross-sectional view of the fabricated microtoroid-based biosensor.^76^ (a) SEM image of the UHQ (ultra-high quality) microtoroid optical resonator. Reprinted with permission from Armani *et al.*, Science **317**, 783 (2007). Copyright 2007 American Association for the Advancement of Science.^76^ (b) A finite element model of a 4-mm minor diameter microtoroid resonator surrounded by water. Note that part of the field leaks into the environment (white arrow). This interaction between the whisper gallery mode and the environment provides ultrasensitive detection. Reprinted with permission from Armani *et al.*, Nature **421**, 925 (2003). Copyright 2003 Springer Nature.^77^ (c) Measured lifetime of 43 ns^77^ corresponds to an inherent Q of ∼1.25 × 10^8^. (d) Microtoroid with the quality of ∼1.83 × 10^8^ and use of IL-2 solutions can provide a dose response of 10^−19^–10^−6^ M and a working range of 5 aM–1 *μ*M.

Microtoroids are resonators with a Q of >10^8^ and a small mode volume that can be fabricated on silicon using standard microelectronics techniques.^76^ However, microtoroids need to be strictly aligned with the tapered fiber waveguide to achieve high coupling and cannot meet our needs for high-throughput multiplexing sensing. Armani *et al.*^76^ demonstrated the possibility of detecting unlabeled single molecules and higher concentrations on a single platform [Figs. 7(a) and 7(b)]. According to reports, the quality of performing planar lithography is ∼1.83 × 10^8^. The authors reported that by using the interleukin 2 (IL-2) solution, the microring sensor can provide a dose response of 10^−19^–10^−6^ M and a working range of 5 aM to 1 *μ*M. In another report for a microtoroid with a diameter of 90 *μ*m, the authors reported^77^ that a measurement lifetime of 43 ns corresponds to an inherent quality factor of 1.25 × 10^8^ [Figs. 7(c) and 7(d)].

#### 3. Photonic crystal

**FIG. 8.**
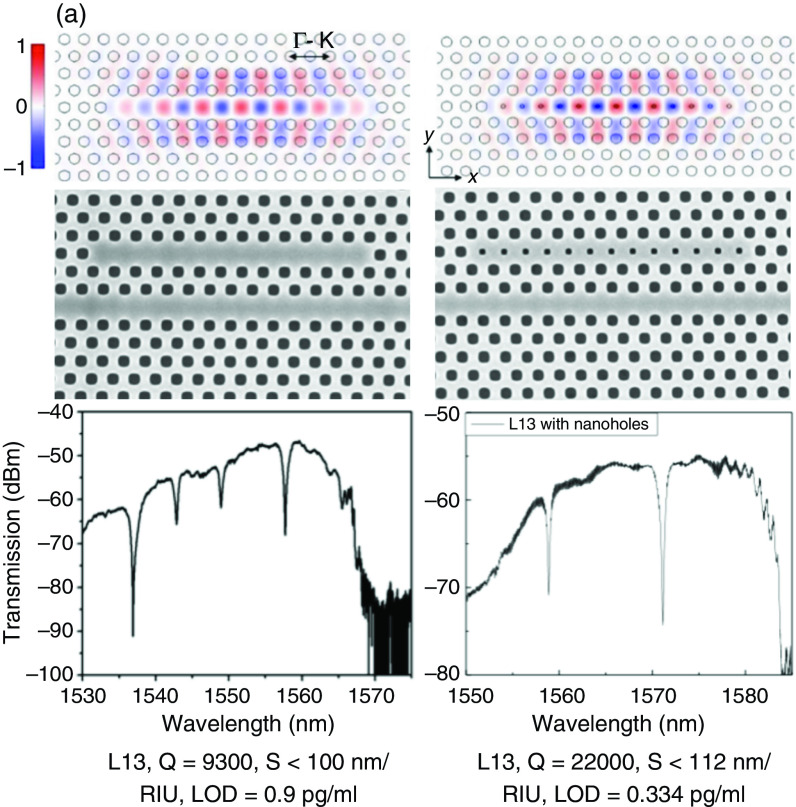
2D PhC microcavity biosensors. (a) Slow light L13 PhC cavity for enhanced sensitivity.^84^ Reprinted with permission from Lai *et al.*, Appl. Phys. Lett. **102**, 041111 (2013). Copyright 2013 AIP Publishing.^84^ (b) Nano holes–assisted high-sensitivity L13 PhC cavity for plasma protein detection of pancreatic cancer.^78^ The Q, S, and LOD performances are annotated below each picture. Note that there is a trade-off between sensitivity and LOD.

For microcavity photonic crystal (PhC) sensors, we mainly use 2D PhC biosensors, which have the advantages of design flexibility, compact size (surface area of about a few square micrometers), and strong light interaction with the analyte of interest. As shown in Figs. 8(a) and 8(b), Yang *et al.* recently confirmed the work of practical pancreatic cancer detection by using nanopore-assisted high-Q (22 000) and high-S (112 nm/RIU) L13 PhC cavities.^78^ The detection results showed that a concentration of 0.334 pg/mL (8.8 fM) pancreatic cancer biomarker was successfully detected in patient plasma samples, which is 50 times more diluted than conventional enzyme-linked immunosorbent assay (ELISA). To go farther, by designing and developing multimode interference (MMI) separators,^52,79–83^ high-throughput and multiplexed biosensor arrays have been proposed and demonstrated [Figs. 8(a) and 8(b)].^52,79–81^ The integrated scheme and array methods proposed and proven improve the multiparameter and multifunction detection capabilities of the sensor and can be used in practical diagnostic applications.

**FIG. 9.**
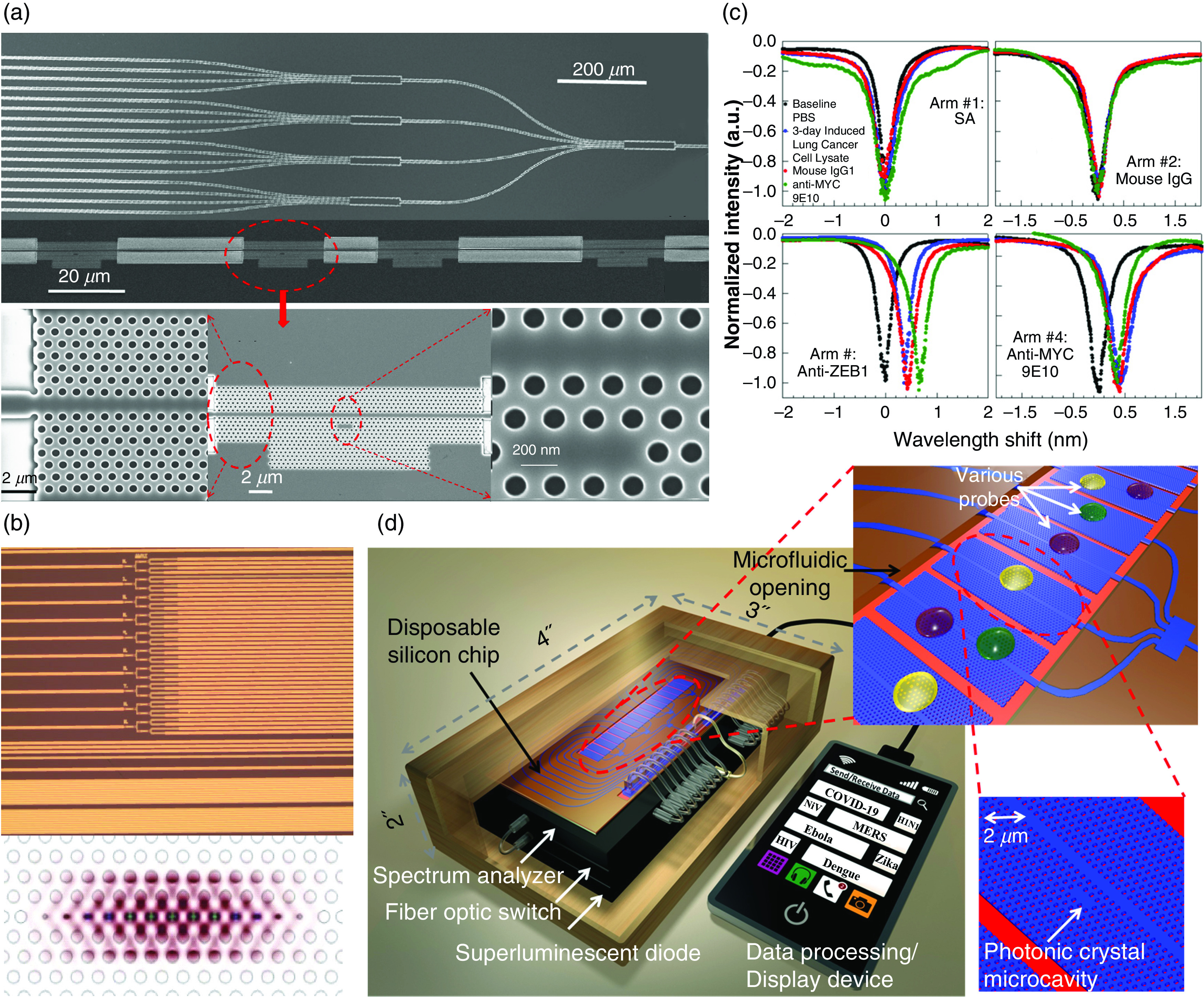
Integrated sensor array for high-throughput detection.^52^ (a) Multiplexed 1 × 4 MMI power splitter that splits an input light into 16 optical paths, each with photonic crystal microcavity sensors for 64 sensors in total. (b) Top: Microscope image of foundry-fabricated silicon photonic crystal sensor devices. Bottom: Highly confined electric field in a photonic crystal microcavity for enhanced analyte sensitivity. (c) Multiplexed, simultaneous, specific detection of ZEB1 in lung cancer cell lysates with four arms of the MMI derivatized with bovine serum albumin, isotype-matched control mouse immunoglobulin G1 (IgG1), anti-ZEB1 antibody, and anti-MYC 9E10 antibody. (d) Comprehensive PCW detection platform. a.u., arbitrary units; PBS, phosphate-buffered saline.

Our team has extensively described the functionalization of silicon surfaces using various probe biomarkers^52^ and their use in the detection of specific conjugated biomarkers using our silicon photonic crystal microarray structure. Previously, we demonstrated a multimode interference coupler architecture [Figs. 9(a) and 9(b)] that shows the series and parallel integration of 64 sensors on the silicon chip. Multiplexed sensing with specificity of lung cancer cell line lysates was demonstrated. We have also demonstrated experimentally that the silicon photonic crystal sensor chips can be fabricated in a commercial foundry for high-volume manufacturing. For COVID-19 testing, the silicon chip manufacturing process and sensor functionalization process will be the same as before.

**FIG. 10.**
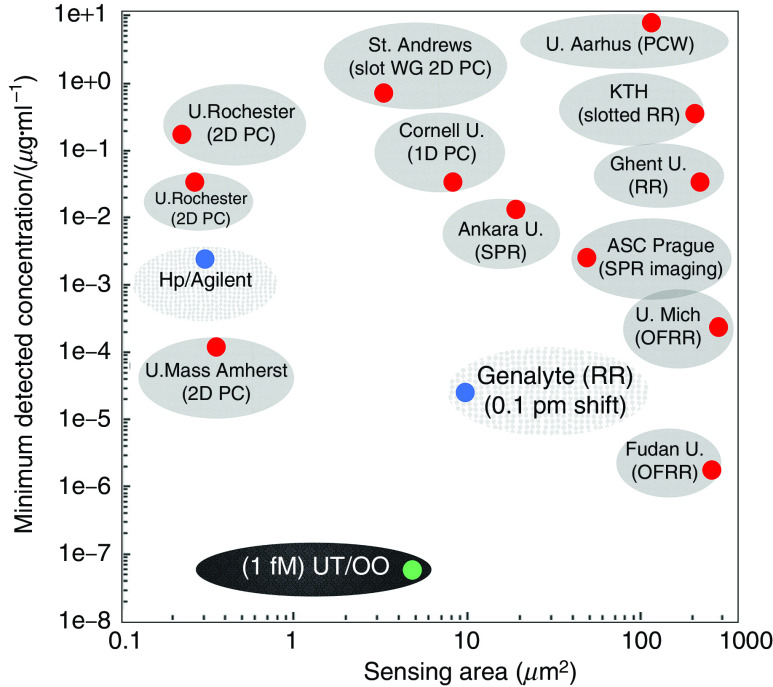
Comparison of minimum experimentally demonstrated detection limits vs other label-free optical platforms as a function of sensing area on chip. UT/OO has detected 67 fg/ml (1 fM), which is the lowest detected concentration reported.^97^ UT/OO is in reference to published works jointly by UT, Austin, and Omega Optics, Inc. ASC Prague, Academy of Sciences of the Czech Republic; BIND, biomolecular interaction detection; KTH, KTH Royal Institute of Technology; LCR, liquid crystal sensors;^96^ OFRR, opto-fluidic ring resonator;^94,95^ PC, photonic crystal;^52,64,65,73–75,79–89^ RR, ring resonator;^74,90–92^ SPR, surface plasmon resonance;^45,93^ U., University; U. Mich, University of Michigan; WG, waveguide.

Label-free microarrays are particularly exciting because they simplify biochemistry significantly when probe–target binding conjugations can be studied without steric hindrance associated with fluorescent or radioactive tags. In Fig. 10, we compare our photonic crystal microarray approach with other research performed using PhCs and show that our microarray has the highest sensitivity to small changes in concentration. It summarizes sensitivities and detection limits demonstrated in our system compared to other label-free methods, including surface plasmon resonance (SPR), optofluidic ring resonator (OFRR), ring resonator (RR), and PhC devices, as a function of sensing area. Sensitivities of PhC microcavity structures demonstrated at Omega Optics (OO) and The University of Texas (UT), Austin, are shown. It is important to note that the PhC sensors with the lowest detection limit of 67 fg/ml (1 fM) was the result of several carefully optimized processes. First, the L13 cavity with high group index was selected based on a series of comparisons, such as with L21, L55 cavities, and optimized (coupling length between the cavity and the guiding waveguide and the waveguide width) to achieve high Q up to ∼10^4^ in experiments. Furthermore, low refractive index modulators (air holes here) were optimized (mainly the radius) and added to the cavity area to enhance the light–matter interaction and, thus, the sensitivities (because of high filling factor). Thus, the minimum detectable concentration could be obtained from the optimal dependence on Q, group index, and filling factor. In fact, compared with the various SPR- or RR-based sensors, the most important advantages of PC sensors are the minimized sensing areas and the ultra-high-Q performances, which significantly contribute to the practical applications of point-of-care biosensors, fulfilling the requirements of miniaturization, microdosage, and low detection limit. It is worth mentioning that as far as the design and fabrication complexities are concerned, the SPR sensors are the simplest, and the RR sensors are simpler than PhC sensors.

Our photonic crystal microcavity not only has high sensitivity and low detection limit but also can achieve dense integration of sensors due to its small geometric size. In Table I, we compare our proposed PhC microarray platform with other optical biosensing methods capable of detecting SARS-CoV-2. We compared the limit of detection advantages of our proposed platform and show that our platform can provide comparable high sensitivity to other methods. The relatively big size of the S protein trimer in SARS-CoV-2 compared to other detected analytes in these works dictate the applicability of the Table I methods as COVID-19 biosensors.^64,74,78,84,86–90,92–94,96,98–100^

**TABLE I.** Summary of optical biosensors capable of detecting COVID-19 vs our work based on the requirements.

Technique	Ring resonator	Liquid crystal	Surface plasmon resonance	Photonic crystal	Our work	COVID-19 biosensor requirement
Limit of detection	0.1 pM	15 fM	0.22 pM	1 pM	1 fM	< pM
Target biomarker	Dopamine	BSA^a^	miRNA	Biotin	Advidin	S protein
Target biomarker size	70 kDa	66 kDa	6.5 kDa	0.2 kDa	67 kDa	600 kDa
Ref.	96	95	98	101	97	44

^a^ BSA, bovine serum albumin.

## III. PLASMONIC OPTICAL BIOSENSORS

A plasmon can be described as a collective oscillation of a free electrons or a quantum of plasma oscillation. Propagation of electromagnetic waves along the surface of a metallic surface or surface plasmons (SPs) can be understood as a strong interaction between conduction electrons of the metallic surface and electromagnetic waves, which leads to resonance modes trapped on the surface, also known as SPRs.^24,45,50^ SPR propagation along the conductor surface produces a charge density distribution, which enhances the light–matter interaction on the nanoscale. Such enhancements, so-called “hot spots,” that occur at the interface between a dielectric and metallic surface offer the higher sensitivity for plasmonic biosensors. Many types of optical biosensors based on plasmonic platforms have been studied as fascinating candidates for biomedical and chemical sensors.^26,33,51,102–112^ The selectivity of the plasmonic-based biosensor can be achieved by using immobilization of the various bioreceptors. Depending on the target analytes, specific bioreceptors can be selected to be immobilized on the surface of the sensor and react or bind only to its counterparts. Based on the device configurations, plasmonic biosensors can be divided into two groups: SPRs and localized SPRs (LSPRs).

### A. SPR sensor

**FIG. 11.**
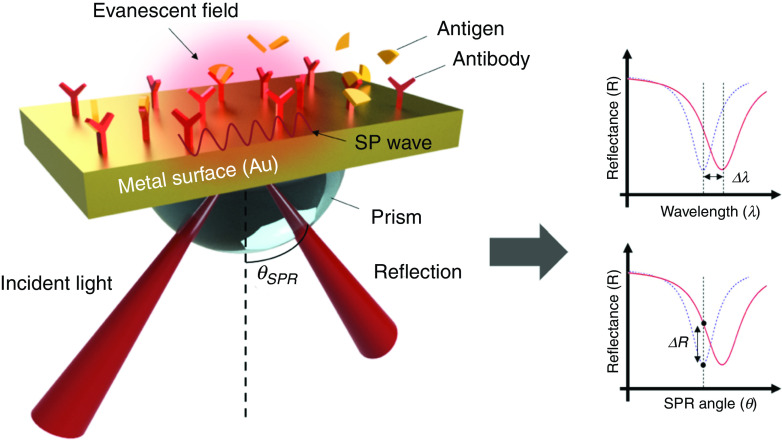
Schematic illustration of the standard SPR-based biosensor configuration.

Fundamentally, when the phase matching condition between the incident light and the SP wave guided along the metal/dielectric interface is reached, the incident light can be coupled to the surface-guided mode. Note that the resonance condition between the incident light and the conductive electrons at the metal–dielectric interface with a fixed angle of incidence is only achieved at a specific wavelength. The guided light will be absorbed by the conducting electrons that resonate, which will significantly reduce the reflected light at that particular wavelength. Therefore, once the target molecule is attached to the functionalized metal film, the refractive index changes, causing a shift in the resonance wavelength. Consequently, SPR angle alteration can be characterized as the main sensing mechanism. Several coupling methods have been proposed, including a grating coupler, a waveguide coupler, and a prism coupler, but the prism coupling method has been used as a standard configuration based on the Kreichman configuration.^112^ Figure 11 shows the schematic of the conventional SPR sensor configuration.

The wavevector of the evanescent field of the incident electromagnetic wave propagating along the prism–metal interface is shown in the following equation:^102,112^

$$k_{in}=\frac{2\pi }{\lambda }n_{p}\text{ sin}\left(\theta \right),$$ (6)

where $n_{p}$ is the refractive index of the prism, λ is the wavelength of the incident light, and θ is the incident angle.

The wavevector of the SP wave propagating along the metal/dielectric interface is as follows:^113,114^

$$k_{SP}=\frac{\omega }{c}\sqrt{\frac{{n_{D}}^{2}{n_{M}}^{2}}{{n_{D}}^{2}+{n_{M}}^{2}}},$$ (7)

where ω is the angular frequency of the wave, *c* is the speed of light in vacuum, and $n_{M}$ and $n_{D}$ are the refractive indices of the metal and dielectric, respectively. As mentioned before, the resonance condition is met when $k_{in}=k_{SP}$, so we can calculate the SPR angle in the following equation:

$$\theta_{\mathrm{SPR}}={\text{sin}}^{-1}\left(\frac{1}{n_{p}}\sqrt{\frac{{n_{D}}^{2}{n_{M}}^{2}}{{n_{D}}^{2}+{n_{M}}^{2}}}\right).$$ (8)

The sensitivity of the SPR devices is determined by the resonance shift with respect to the change of the refractive in the absence and presence of the target analyte,^113–116^

$$S=\frac{\Delta \lambda }{\Delta n},$$ (9)

where Δλ is the resonance wavelength shift and Δn is the change of bulk refractive index, including the target analyte.

### B. Localized SPR sensor

**FIG. 12.**
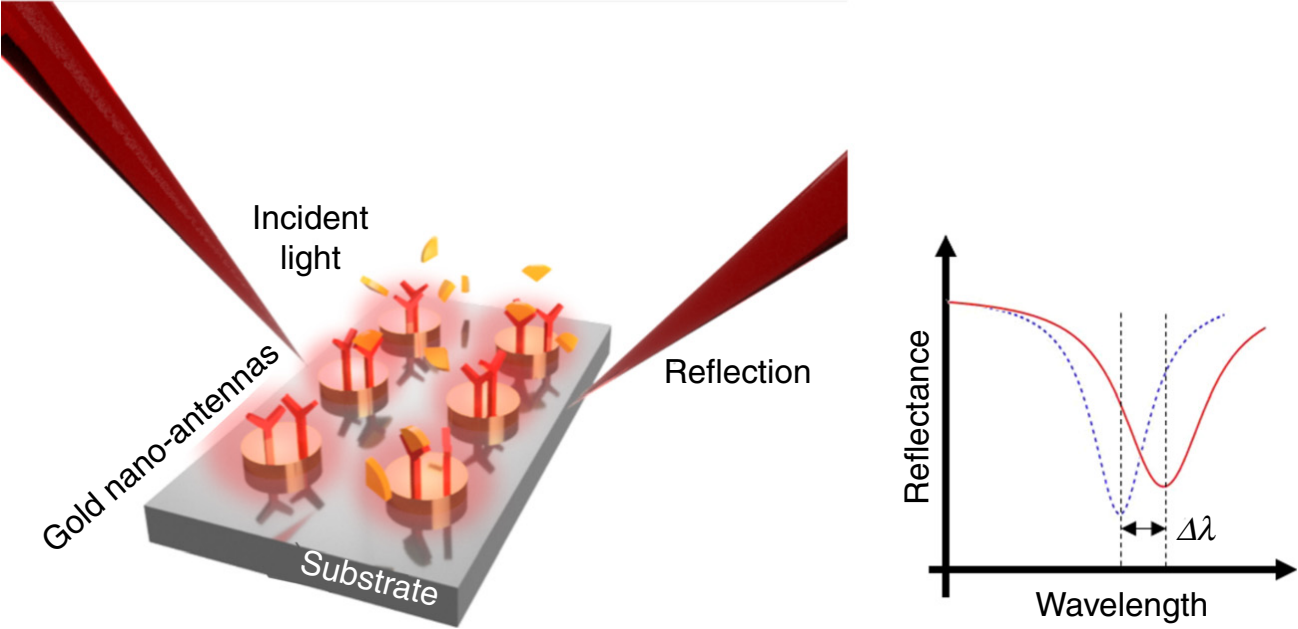
Schematic illustration of the resonance wavelength shift sensing based on LSPR sensor configuration.

On the other hand, nanostructures in conductive thin films are among the essential building blocks of LSPR plasmonic biosensors (see Fig. 12). These nanoscale geometric/periodic lattice factors bring huge advantages over conventional SPR devices. Contrasted with SPR occurring along the propagation surface, the attenuation length of the local electromagnetic field is much shorter. These strict restrictions, with a shorter subwavelength structure, can achieve ultra-low-mode volume resonance, making it sensitive to environmental refractive index changes, which are particularly helpful for the detection of tiny biological molecules. Also, an incident light can be directly coupled to the SP wave on the conductive structures without any external couplers, e.g., prisms or gratings, which ameliorates the complexity of the entire system and enables sensor miniaturization^117^ and absorbance, transmittance, and reflectance-based sensing.^43,106–108,118–120^

Moreover, LSPRs can be utilized for various types of resonance modes and detection methods, including surface-enhanced Raman spectroscopy (SER),^111,112^ photoluminescence/fluorescence,^55,110^ and mid-infrared spectroscopy,^105^ by tuning the resonance wavelength for a specific light–matter interaction. Table II shows the comprehensive comparison between conventional SPR and LSRP biosensors.

**TABLE II.** Comparison between SPR and LSPR sensors.

	SPR	LSPR
Sensing distance	∼1000 nm	∼10 nm (tunable)
Coupling components	Required (prism, gratings, etc.)	Not required
Sensor miniaturization	Limited	Effective
Detection methods	Angle shift, wavelength shift	Wavelength shift, extinction, scattering, imaging, SEIRA,^a^ SER, fluorescence, photoluminescence
Label-free detection	Yes	Yes
Response time (real-time detection)	<10^3^ s	<10^3^ s
Specificity	Achieved by surface functionalization	Achieved by surface functionalization; SEIRA offers the identification of molecule chemical bonds
Multiple microfluidic channel compatibility (parallel detection)	Limited	Yes

^a^ SEIRA, surfaced-enhanced infrared absorption.

#### 1. Resonance shift sensing

Figure 12 shows the basic configuration of the LSPR device based on resonance shift sensing. The metal nanostructure on the dielectric substrate is used as a resonator, and due to the aforementioned advantages, the sensitivity can be further improved compared with the conventional SPR resonance shift device. The sensitivity and the figure of merit (FOM) of LSPR resonance shift sensors follows the same definition of SPR as in Eq. (10). Another important performance factor of resonance-based sensors is the Q factor, which is defined as^121,122^

$$Q=\frac{\lambda_{o}}{\mathrm{FWHM}},$$ (10)

where $\lambda_{o}$ and FWHM are the wavelength and full-width half maximum of the resonance peak, respectively. To enhance the sensing performance, a higher Q value is desirable because sharper peaks with high Q values are much easier to detect. Considering all these factors, the inherent limit of detection (ILOD) of the resonance displacement sensing device can be defined as follows:^69,123^

$$\mathrm{ILOD}=\frac{\lambda_{o}}{Q\cdot S},$$ (11)

which indicates that both the higher sensitivity (S) and Q factor are required to minimize the limit of detection of the sensors.

Although these so-called hot spots provide higher sensitivity for LSPR biosensors, their performance is greatly limited due to the basic limiting factors of ohmic losses in metal surfaces. In other words, compared to other photonic biosensors, the absorption loss in the conductive nanocavity leads to a low Q value. To enhance the sensing performance (represented by LOD), researchers have achieved a higher sensitivity and Q factor by using advanced materials^142,143^ or optimizing the geometry of metamaterials.^136,140^

#### 2. Plasmonic perfect absorber

**FIG. 13.**
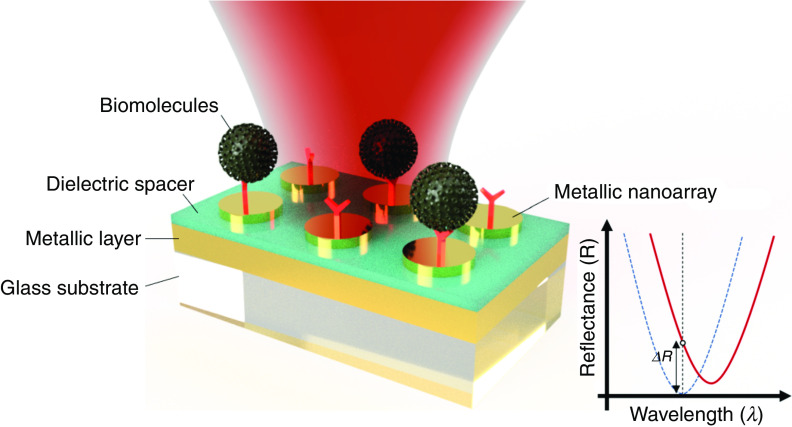
Schematic illustration of LSPR perfect absorber–based sensor.

On the other hand, the concept of a plasma perfect absorber (PPA) sensor was introduced to overcome this intrinsic limiting factor.^124–128^ Figure 13 shows that the typical configuration of a PPA sensor consists of periodically arranged metallic nanoantennas (metamaterial) on top and a thin metallic “mirror” layer on the bottom separated by a dielectric spacer.^126^ The basic concept is to have a perfect absorbance at the operating wavelength and make a “zero” transmittance by maximizing the metamaterial losses; in other words, the losses provide an advantage in the PPA sensors. In this structure, most of the incident light at the operating wavelength is absorbed by the top nanoantennas operating as a resonator through impedance matching, and the metallic bottom layer acts as a mirror to eliminate the transmittance. As a result, the reflectance of light can be characterized for sensing as in Fig. 13, and the ${\mathrm{FOM}}_{\mathrm{PPA}}$ is defined as follows:^127^

$${\mathrm{FOM}}_{\mathrm{PPA}}=\left|\frac{dI\left(\lambda_{o}\right)/I(\lambda_{o})}{dn(\lambda_{o}).}\right|,$$ (12)

where $dI\left(\lambda_{o}\right)/I(\lambda_{o})$ is the relative intensity change of reflected light at a fixed resonance wavelength $\lambda_{o}$, which is induced by a refractive index change $dn(\lambda_{o})$.

Moreover, it has been shown that the perfect absorption (>99%) of incident light at working wavelength can remain over a wide incident angle and be insensitive to the polarization (TE/TM) of incident light.^127^

#### 3. Surface-enhanced infrared absorption spectroscopy

Another important detecting method using LSPR is surface-enhanced infrared absorption (SEIRA) spectroscopy. Based on molecular absorption spectroscopy and the fundamental vibrational–rotational transitions of chemical bonds in the wavelength of 3–20 *μ*m, mid-infrared (MIR) absorption spectroscopy has been studied vigorously for label-free detection and identification of molecules in the optical sensor domain. In particular, unlike the near-infrared (NIR) wavelength region, the molecular fingerprint region (700–1500 cm^−1^) in MIR wavelengths contains many absorption bands related to bending and stretching of chemical bonds (such as −C−C−, −C−O−, −C−N−, etc.) that allow the unique identification of biomolecules with high sensitivity and specificity.

**FIG. 14.**
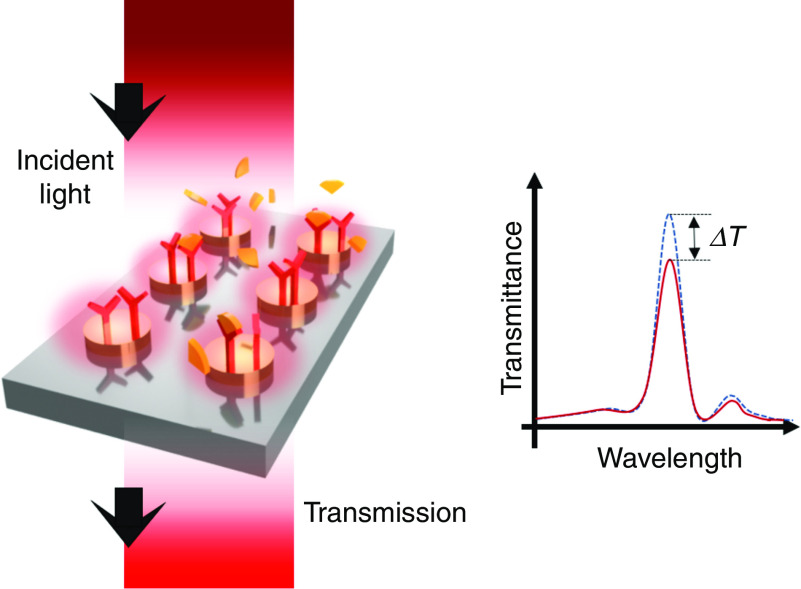
The intensity change of transmitted light–based LSPR sensor, typically adapted for mid-infrared absorption spectroscopy.

Among many types of optical-based molecular absorption spectroscopy platforms, SEIRA spectroscopy for LSPR devices has shown great promise for detecting a thin layer of surface-bound nanomolecules due to its tight confinement of surface plasmons on metallic nanostructures, which can significantly enhance the IR absorption of small molecules. Figure 14 shows the typical configuration of SEIRA spectroscopy using an LSPR sensor. When the plasmonic resonance peak generated by the metallic nanoantennas is matched with the fundamental vibration signatures of chemical bonds in the biomolecules, the coupling of molecular transitions with the LSPR field on the surface allows significant absorption of the corresponding wavelength, so the decrease in transmitted light can be characterized as a sensing result. To obtain more intense IR absorbance, researchers have shown various nanostructures, including nanorod antennas,^129^ coaxial nanogaps,^130^ and nanocavities,^131–133^ to enhance the optical confinement and field enhancement of MIR light.

**FIG. 15.**
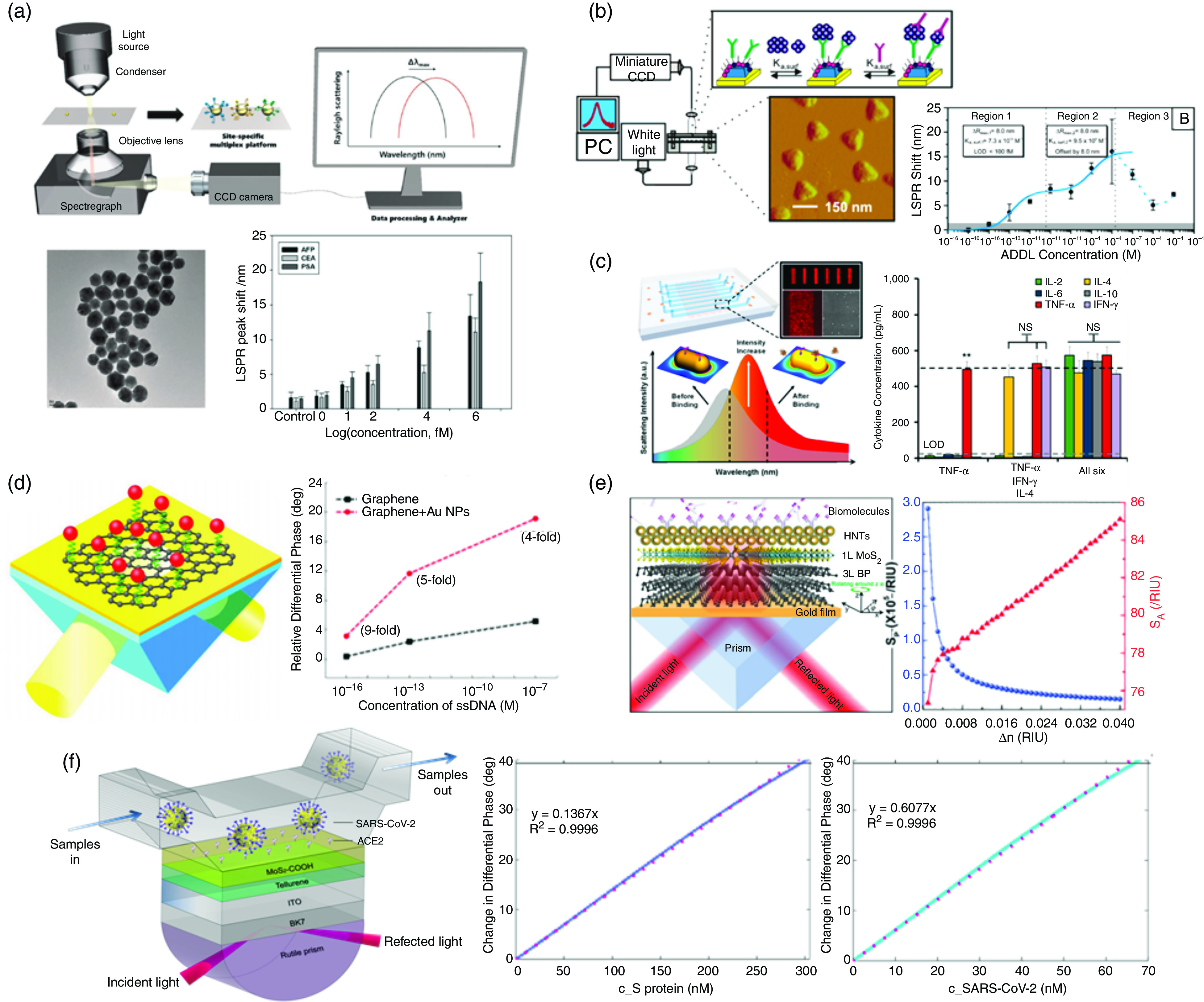
Various SPR and LSPR biosensing applications. (a) Multiplex biosensor for cancer biomarker detection based on the resonance shift of LSPR single gold nanoparticles; LOD of 91 fM, 94 fM, and 10fM for AFP, CEA, and PSA, respectively.^43^ Reprinted with permission from Lee *et al.*, Biosens. Bioelectron. **74**, 341 (2015). Copyright 2015 Elsevier.^43^ (b) Alzheimer disease biomarkers from clinical samples using surface-confined Ag nanoparticles and sandwich assay; LOD of < 100 fM for ADDLs.^108^ Reprinted with permission from Haes *et al.*, J. Am. Chem. Soc., **127**, 2264 (2005). Copyright 2005 American Chemical Society.^108^ (c) Multiplex serum cytokine immunoassay using gold nanorod microarray conjugated with antibodies to detect cytokine species in LOD of 5–20 pg/ml (∼0.38–1.54 pM).^118^ Reprinted with permission from Chen *et al.*, ACS Nano, **9**, 4173 (2015). Copyright 2015 American Chemical Society. (d) Ultrasensitive graphene–gold metasurface SPR sensor with LOD of 1 aM.^134^ (e) Ultrasensitive SPR sensor based on halloysite nanotubes/MoS_2_/black phosphorous hybrid surface. Reprinted with permission from Jia *et al.*, J. Mater. Chem. C **7**, 3843 (2019). Copyright 2019 Royal Society of Chemistry.^135^ (f) Phase modulation plasmonic biosensor for sensitive detection of SARS-CoV-2 and its S glycoprotein using 2D van der Waals heterostructures.^181^ Reprinted with permission from Peng *et al.*, New J. Phys. **22**, 103046 (2020). Licensed under a Creative Commons Attribution (CC BY) license.^181^ a.u., arbitrary units; PC, personal computer.

Taking all these device structures and detection methods into account, a number of nanostructure designs have been studied and optimized to apply the plasmonic resonance sensor for the detection of various bioanalytes with high sensitivity, which can be potentially applied for SARS-CoV-2 sensing applications. As shown in Fig. 15(a), Lee *et al.* showed a multiplex biosensor for cancer biomarker detection based on the resonance shift of the LSPR single gold nanoparticles (AuNPs); the selective sensing results with LODs of 91 fM, 94 fM, and 10 fM for the α-fetoprotein (AFP), carcinoembryonic antigen (CEA), and prostate specific antigen (PSA) analytes, respectively, are reported by antibody–antigen binding.^43^ Haes *et al.* reported the detection of Alzheimer disease biomarkers from clinical samples [Fig. 15(b)] using surface-confined Ag nanoparticles and sandwich assay; the LOD of < 100 fM for amyloid β-derived diffusible ligand (ADDL) detection with the specific anti-ADDL antibodies is reported by the LSPR-induced wavelength shift.^108^ Chen *et al.* reported multiplex serum cytokine analysis by immunoassay that was enhanced using nanoplasmonic biosensor microarrays [Fig. 15(c)]. Periodically arranged gold nanorod microarray conjugated with corresponding antibodies of each cytokine species [IL-2, IL-4, IL-6, IL-10, (IFN-γ), and (TNF-R)] results in LODs of 5–20 pg/ml from a 1-*μ*l serum sample within 40 min.^118^ Integration of graphene with an SPR sensor, as reported by Zeng *et al.*, has shown ultrasensitive sensing [Fig. 15(d)].^134^ They reported an LOD of 1 aM for 7.3-kDa 24-mer single-stranded DNA (ssDNA). Moreover, an ultrasensitive SPR sensor based on halloysite nanotubes (HNTs)/MoS_2_/black phosphorous (BP) atomic layers on gold films have been introduced by Jia *et al.*, with the angular and phase detection sensitivities up to S_A_ = 77.1 RIU^−1^ and S_P_ = 1.61 × 10^5^ RIU^−1^, respectively^135^ [Fig. 15(e)]. Most recently, a theorical study of a novel phase modulation plasmonic biosensor working in the NIR region, which can be employed for sensitive detection of SARS-CoV-2 and its S glycoprotein, was proposed using 2D van der Waals heterostructures, including tellurene and carboxyl-functionalized molybdenum disulfide (MoS_2_) layers, with transparent indium tin oxide (ITO) film [Fig. 15(f)];^176^ the highest detection sensitivity of 8.41 × 10^4^°/RIU and an excellent linear detection range of 0∼301 nM and 0∼67.9 nM for S glycoprotein and SARS-CoV-2 specimens were explained.

#### 4. SARS-CoV-2 sensing application

**FIG. 16.**
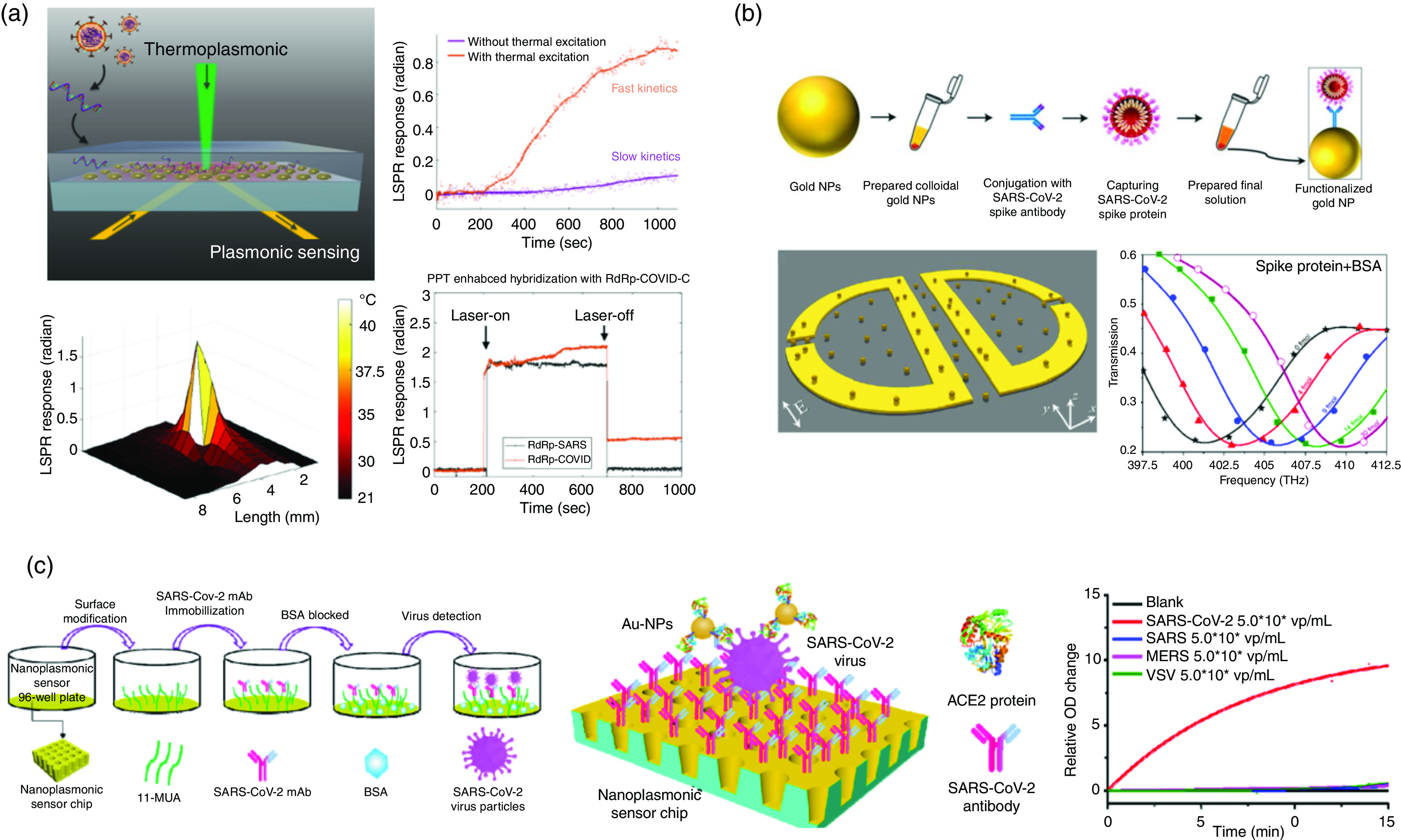
State-of-the-art plasmonic biosensor for SARS-CoV-2 sensing applications. (a) Photothermal-enhanced LSPR biosensor for nucleic acid sequence detection from SARS-CoV-2. The schematic shows that the configuration of the LSPR device consists of gold nanoparticles where the local heat is generated by the thermoplasmonic effect. The graph shows the sensing enhancement by the thermal excitation with LOD of 0.22 pM and the sensing selectivity between the RdRp-SARS and RdRp-COVID hybridization.^33^ Reprinted with permission from Qiu *et al.*, ACS Nano **14**, 5135 (2020). Copyright 2020 American Chemical Society.^33^ (b) A femtomolar-level detection of SARS-CoV-2 spike proteins using toroidal plasmonic metasensors; the schematic shows the visual illustration of AuNP–enhanced toroidal plasmonic metasensors, and the workflow of the developed functionalized AuNPs conjugated with the SARS-CoV-2 antibody and S proteins. The transmission spectra of the THz metasensor device for 30 different concentrations are measured, with LOD of ∼4.2 fmol (0.28 nM).^136^ Reprinted with permission from Ahmadivand *et al.*, Biosens. Bioelectron. **177**, 112971 (2021). Copyright 2021 Elsevier.^136^ (c) SARS-CoV-2 nanoplasmonic sensors using an array of Au-TiO_2_-Au nanocup structures. The change of the transmission optical density (OD) is measured to quantify the virus concentration linearly in the range of 103–106 vp/ml; the minimum detection limit of as few as 30 vp (0.03 fM) is reported.^137^ Reprinted with permission from Huang *et al.*, Biosens. Bioelectron. **171**, 112685 (2021). Copyright 2021 Elsevier.^137^ BSA, bovine serum albumin; MUA, mercaptoundecanoic acid; VSV, vesicular stomatitis virus.

Here, we review the most up-to-date advances, especially for the coronavirus sensors in the plasmonic domain, and introduce well-established plasmonic SARS-CoV-2 biosensing systems. Researchers have demonstrated that using SPR/LSPR-based sensors and corresponding binding biological receptors can effectively and selectively detect coronavirus.^26,102,104^ Moreover, several researchers have already reported experimental SARS-CoV-2 sensing results as shown in Table III and Fig. 16.^33,120^

**TABLE III.** Summary of applications of various plasmonic biosensors for the coronavirus family.

Analyte	Detection method	Material	Functionalization	Limit of detection	Ref.
SARS-CoV-2	PPT/LSPR	2D AuNI	DNA hybridization	0.22 p M	33
SARS-CoV-2	SPR	AuNP–enhanced plasmonic metasurface	Antigen–antibody binding	30 virus particles (0.03 fM)	136
HCoV,^a^ MERS-CoV	LSPR	Array of carbon electrodes (DEP)^b^ modified with gold nanoparticles	Antigen–antibody binding	HCoV −0.4 pg ml^−1^ (2.7 fM), MERS-CoV −1.04 pg ml^−1^ (6.9 fM)	102
SARS-CoV	ELISA/LSPR	PMMA optical fiber/AuNPs	Antigen–antibody binding	1 pg ml^−1^ (22 fM)	26

^a^ HCoV, human coronavirus.

^b^ DEP, dielectrophoresis.

A recent article reported the dual-functional plasmonic photothermal biosensors for SARS-CoV-2 detection [Fig. 16(a)].^33^ The authors demonstrated a highly sensitive, fast, and reliable SARS-CoV-2 virus detection capability by integrating the plasmonic photothermal (PPT) effect and conventional LSPR sensing transduction on a single gold nanoisland (AuNI) chip. The two-dimensional AuNIs functionalized with cDNA receptors (RdRp-COVID-C) can perform a selective detection of the RdRp-COVID through DNA hybridization, and a LOD down to the concentration of 0.22 pM was reported. Ahmadivand *et al.* demonstrated a femtomolar-level detection of SARS-CoV-2 spike proteins using toroidal plasmonic metasensors [Fig. 16(b)].^136^ Based on the unconventional features of the toroidal metasensors, which can focus down to the incident electromagnetic radiation within a tiny hot spot, these metamolecules support resonances that possess much higher sensitivity to the refractive index perturbations in the surrounding media. Moreover, to improve the binding properties, functionalized colloidal AuNPs conjugated with the respective antibody and captured the S proteins present in the sample. The resonance shifts for diverse concentrations of the S protein is monitored with the LOD of ∼4.2 fmol (0.28 nM). Other research with a similar concept but different structures was reported using nanoplasmonic resonance sensors.^138^ The low-cost nanoplasmonic sensor chips consist of a Au-TiO_2_-Au nanocup array that allow observation of the plasmon resonance wavelength and intensity change on the virus-capturing event by transmission light spectroscopy without any external coupling optics. The sensitivity is enhanced by applying a AuNP-enhanced sandwich plasmonic resonance immunoassay method, as shown in Fig. 16(c), and the authors demonstrated the minimum detection of 30 virus particles (vp) in one step within 15 min with specificity.

Furthermore, several researchers reported that the sensitivity and the signal-to-noise ratio (SNR) of conventional ELISA or fluorescence-linked immunosorbent assay (FLISA) tests can be significantly improved by applying the “add-on” plasmonic particles without altering their workflow.^26,55^ As the ELISA test is widely used for precise SARS-CoV-2 detection,^26^ plasmon enhanced ELISA/FLISA tests can be applied to COVID-19 sensing as well.

Although the aforementioned SARS-CoV-2 sensing applications^33,120^ have shown great specificity and performance of sensitive and selective specificity and sensitivity up to a few fM, a huge potential for more sensitive, accurate, and fast on-chip sensing with a less complex system still remains in the LSPR biosensor domain due to smaller sensing volume and electromagnetic decay length. For example, the sensing systems in Fig. 16(a) require the prism coupler to couple the incident light into an SPR device with an accurate incident angle. It requires very sensitive alignment of optical devices, which makes the overall system complex and hard to integrate with sources and detectors. However, as described in Table II, the incident light can be coupled into LSPR sensors directly without external couplers, and this normal incident angle can make the alignment easier, in turn mitigating the complexity of the system and making fully integrated on-chip sensing possible. Moreover, due to the capability of sensor miniaturization through LSPR nanostructures, label-free, real-time, and parallel detection with multiple channels with high specificity is achievable. Furthermore, improving the sensitivity by applying advanced materials like graphene and 2D materials (2DMs) has incited a great interest for various optical biosensor applications. For plasmonic biosensors, the ultrasensitive graphene and 2D material–enhanced SPR devices have been reported [Figs. 15(d)–15(f)], and the experimental sensing result with LOD value approaching 1 aM has been shown.^134,139^ Accordingly, the LSPR biosensors enhanced with advanced materials are anticipated to enable the possibility of a highly sensitive, accurate, and fast point-of-care lab-on-chip–integrated sensor with unprecedented high sensitivity and limit of detection of up to sub-fM. A detailed discussion of emerging nanomaterials, such as graphene and graphene oxide, for optical biosensors are described in Sec. V.

## IV. SARS-CoV-2 BIOSENSOR: DESIGN AND IMPLEMENTATION

**FIG. 17.**
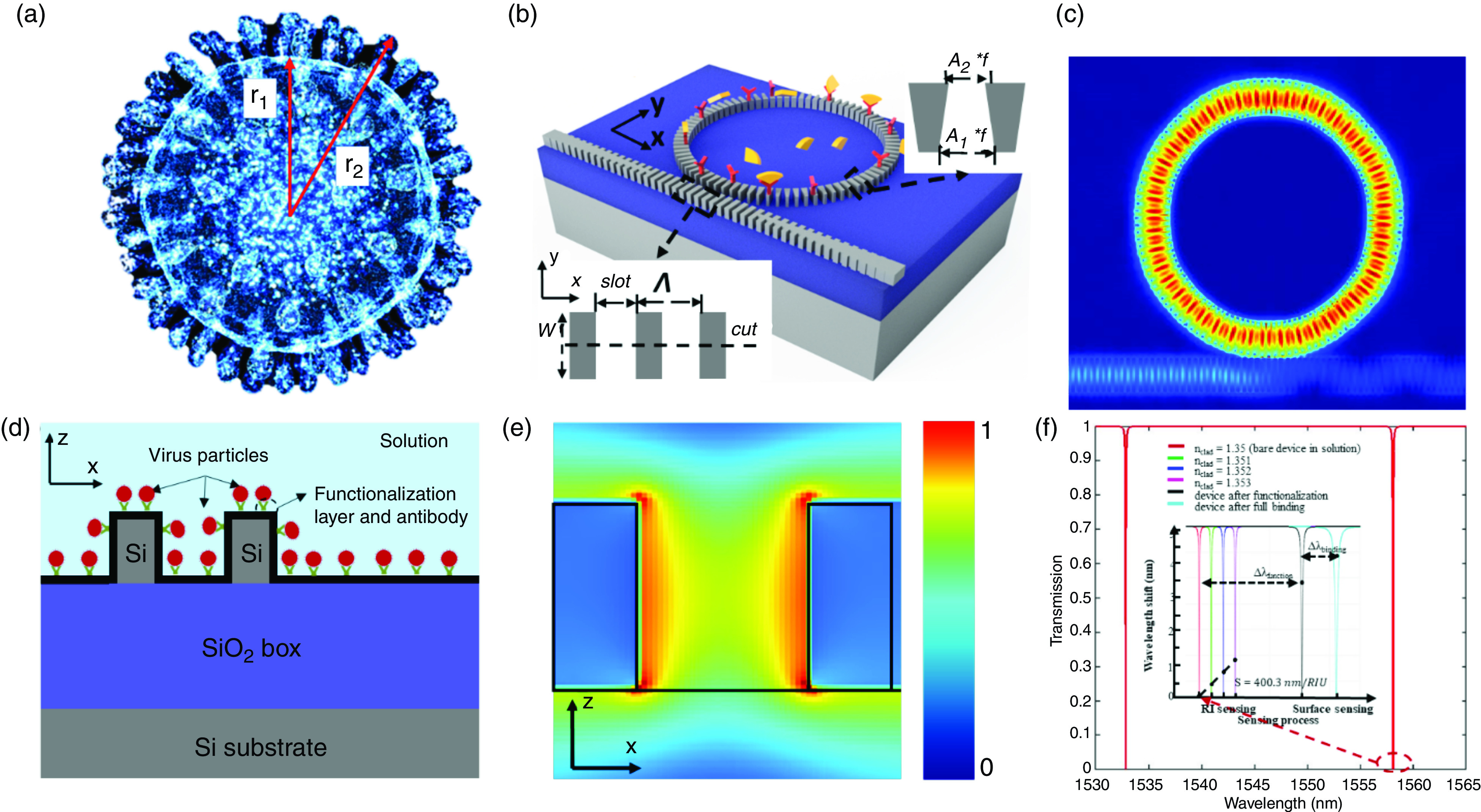
(a) Schematic of COVID-19. (b) Schematic illustration of the SWGR biosensor with a low loss in/outcoupling via lineally tapering the input and the output gratings. (c) Top view of the simulated SWGR biosensor for a fundamental TE mode at 1550 nm. (d) Details of the bonded COVID-19 on the substrate. (e) Simulated intensity distribution of the electric field at 1550 nm. Light penetrates between the slot waveguides, providing better light–matter interaction with the COVID-19 analyte. (f) Calculated transmission spectrum of the proposed device. Inset shows the calculated bulk RI sensing and surface sensing responses of the device, with the bulk RI sensing showing a sensitivity of 400 nm/RIU and surface sensing showing the total wavelength shifts of 3.41 nm and 1.14 nm after the functionalization process and the full-binding process, respectively.

To develop an accurate estimate of a COVID-19 biosensing functioning mechanism, a simulation model first needs to be designed. Here, in a proposed simulation model, COVID-19 is approximated to be a solid sphere core containing RNA covered with a membrane protein with radii of *r_1_* and *r_2_*, respectively [Fig. 17(a)].^50^ Thus, the effective RI of the virus is calculated by taking a volume-weighted sum of the two refractive indices,

$$n_{\mathrm{eff}}=\frac{n_{1}V_{1}}{V_{1}+V_{2}}+\frac{n_{2}V_{2}}{V_{1}+V_{2}}=\frac{n_{1}+n_{2}(\eta^{3}-1)}{\eta^{3}},\;r_{2}=\eta r_{1},$$ (13)

where *n_1_* (*V_1_*) and *n_2_* (*V_2_*) are the total RI of the RNA and the membrane protein volume, respectively. As the RI of the virus is determined mainly by material composition rather than by its geometrical size, *η* is a constant value for the same kind of virions [*η_COVID-19_* = 1.25 average value of several measurements of transmission electron microscopy (TEM) pictures^140^]. We use the SWGR design to simultaneously take advantage of the enhanced binding surface and strong light–substance interaction. As shown in Figs. 17(c) and 17(e), the energy mode is distributed between the gratings as well. To further improve the SWG waveguide functioning in the subwavelength range, the grating period *Λ*, the waveguide width w, and the fill factor are designed to be 230 nm, 1.23 *μ*m, and 0.5 *μ*m, respectively. For the SWGR, the radius is set as 5 *μ*m with the corresponding FSR^141^ of 25 nm at 1550 nm. Here, the simulation system includes a 220-nm silicon top layer with a 3-*μ*m buried oxide (BOX) wafer and a liquid solution, with the refractive index of *n_clad_* of 1.35.^45^ Adopting our previous designs features,^142,143^ we optimized a high-Q SWGR by utilizing a trapezoidal (T) silicon pillar and reducing bending loss by ∼50% compared to a conventional rectangular silicon pillar. We therefore set the SW waveguide width to 0.5 *μ*m (correlated with the fundamental TE mode) and studied the effect of the trapezoidal width. Note that to obtain the lowest bending loss of the T-SWG waveguide, we employed the particle swarm method for the optimization process. Three parameters (w, A_1_, A_2_) are optimized and defined as the width and the tuning factor of the outer and inner filling factor of the SWG, respectively [as shown in the inset of Fig. 17(b)]. Considering the limitations of the design for fabrication, the slot between gratings is preset to be >60 nm; thus, A_1_ and A2 are limited to be (1, 2) and [0, (1–60 nm/*Λ*)/*f*], respectively. At the same time, to keep the SWG working in the subwavelength regime (*Λ* ≪ λ / 2n_eff_), *Λ* is safely set to be 230 nm, and *f* is simply set to be 0.5 with no optimization. Furthermore, to make the SWG waveguide work as a single or few-mode waveguide, the width of the gratings is set to <2 *μ*m. All in all, the ranges of w, A_1_, and A_2_ are set to be (0.5 *μ*m, 2 *μ*m), (1, 2), and (0, 0.522), respectively. The FOM is defined to achieve the lowest bending loss, with a bend radius of 5 *μ*m. Based on the optimization measures taken, we finally achieved a bending loss as low as 0.0279 dB/cm with the optimized (w, A_1_, A_2_) = (1.23 *μ*m, 1, 0.522). By adjusting the coupling gap between the insertion SWG waveguide and the designed SWGR, the Q can be as high as ∼50 000 (the resonance at 1557.6 nm) with a broad FSR of 25 nm, as shown in Fig. 17(a). We also optimized the 10-*μ*m-radius SWGR [not shown in Fig. 17(f)], achieving a loaded Q of ∼75 000 (the resonance at 1552.1 nm) with an FSR of 11 nm with (w, A_1_, A_2_) = (1.23 *μ*m, 1, 0.522), at the same waveguide–ring cross-coupling coefficients. Needless to say, as the quality factor of the ring becomes higher, the fabrication tends to be more challenging. Bulk RI sensitivity [shown in the inset of Fig. 11(f)] in the buffer solution is calculated to be $S_{\mathrm{res}}=\frac{\Delta \lambda_{\mathrm{res}}}{\Delta n_{\mathrm{clad}}}=400\;\mathrm{nm}/\mathrm{RIU}.$ Thus, the instrument detection limit (iDL) can be calculated to be as low as ∼7.5 × 10^−5^ RIU. Note that iDL performances can be further improved by exploiting a larger radius ring or by further achieving the critical coupling condition given the predictable higher Q while making a trade-off between the performance and the sensor size or the resonance peak extinction ratio.

To evaluate the specific sensing ability of the proposed device for COVID-19, surface sensing performance is analyzed by considering the device immersed in buffer solution bonded by several surface layers (generated in the sensing preparation process), including the ∼2–3-nm surface oxide layer, ∼10-nm functionalization layer with bonded antibody (protein layer), and virus particles layer, in the detection process. In simulations, the layers generated during the preparation process are further simplified to be a uniform layer (RI = 1.45) with a thickness of 15 nm, and the bonded virus layer is simplified as a uniform layer with a thickness of 125 nm (the maximum diameter of the COVID-19 virus) [Fig. 17(a)]. It is noted that the equivalent RI of the virus layer (*n_binding_*) depends on the number of bonded virus layers, which is a function of the virus concentration and binding processing time, and is dominated by the concentration in real sensing process with a given binding time. Thus, the SWGR sensing performance can be evaluated by calculating the *n_binding_* response of the device, with *n_binding_* ranging from 1.35 (no binding) to 1.5 (full binding). Simulation results in Fig.17(f) show that the functionalization and the full-binding process induce a shift of 3.41 nm and 1.14 nm, respectively. The obvious simultaneously measurable shifts in the FSR range ($\Delta \lambda_{\mathrm{res}}<\mathrm{FSR}$) and experimental values ($\Delta \lambda_{\mathrm{res}}\gg 1pm$) indicate the promising potentials of the proposed device in detecting the COVID-19 virus or simply as a chemical/biosensor in future practical applications.

## V. INSIGHT INTO GRAPHENE-BASED OPTICAL BIOSENSORS

**FIG. 18.**
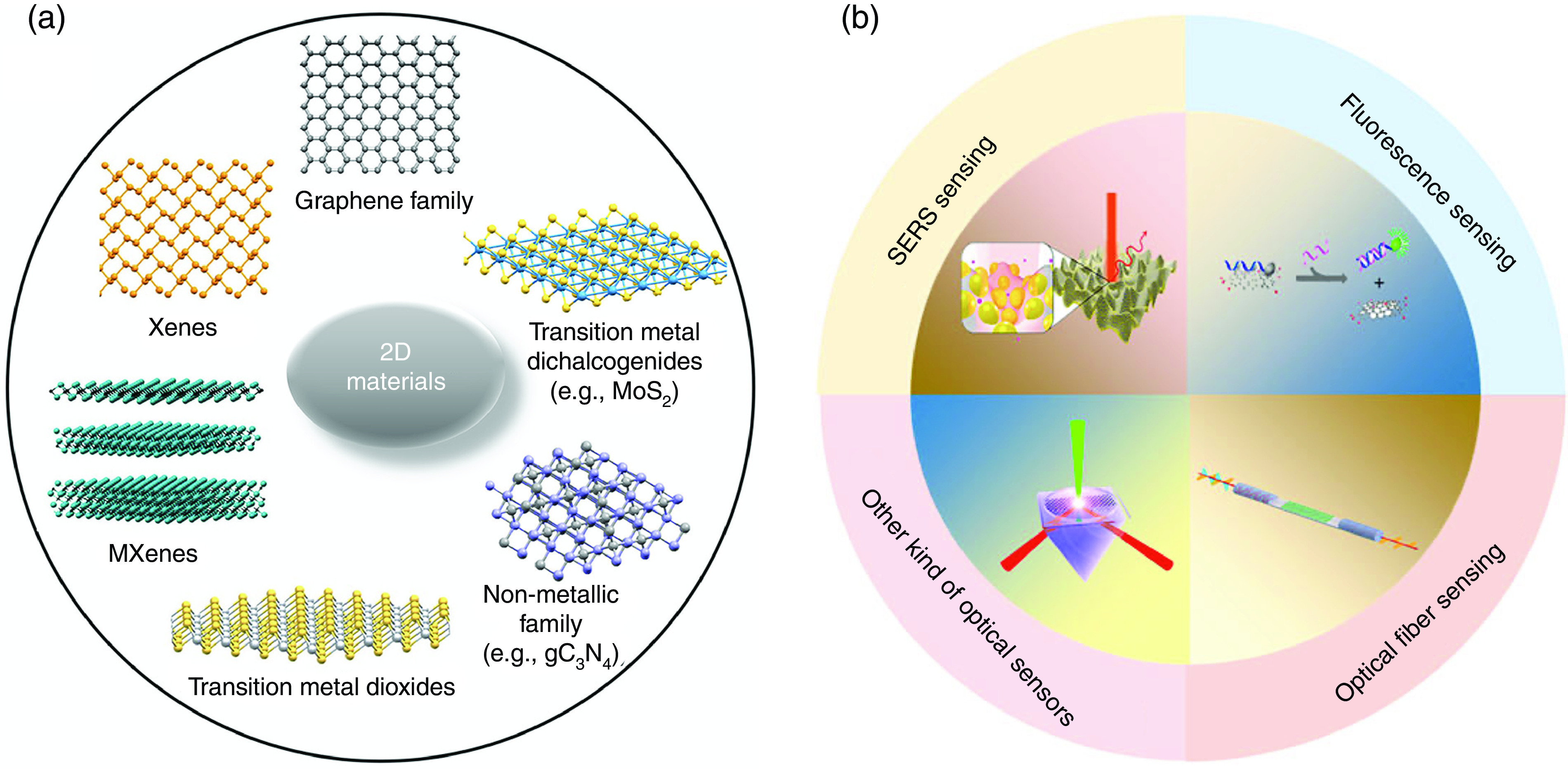
(a) Molecular structure of different families of 2DMs. Reprinted with permission from Menard-Moyon *et al.*, ACS Sens. **5**, 3739 (2020). Copyright 2020 American Chemical Society.^152^ (b) The application of graphene in optical sensing.^154^ Reprinted with permission from Gao *et al.*, Front. Chem. **9**, 615164 (2021). Licensed under a Creative Commons Attribution (CC BY) license.

Ever-increasing advances and developments in the preparation and utilization of nanostructured materials have facilitated the extraction of an unprecedented range of properties from nanomaterials. Biosensing and amplification of targeted samples at extremely low concentrations for detection are among the most intriguing properties.^48^ Two-dimensional materials , as one of the most discussed groups of nanostructured materials, are composed of a variety molecular structures [Fig. 18(a)]. They possess favorable properties such as high surface-to-volume ratio and tunable physicochemical features for detection of biomolecules and various analytes, including virions.^144–149^ Also, a wide range of surface chemistry methods like amino silane and plasma chemistry can be done to immobilize biomolecules on the surface for selective sensing of different target analytes.^150,151^ Therefore, a sensitive 2D material structure with the right biofunctionalization strategy is favored to be employed for a successful early, rapid therapeutic biosensing system.^144,152,153^

Among the 2DM family, graphene possesses a special electron band structure showing high carrier mobility and zero bandgap characteristics, exceptional high-energy transfer efficiency, and large surface area.^46^ Other than that, graphene biosensing properties are easier to exploit due to the sophisticated fabrication process, ease of functionalization, and biocompatibility.^149^

As a result, graphene and its derivatives, like graphene oxide (GO), have been used in various biomedical areas, including but not limited to DNA sequencing, tissue engineering, stem cell research, and biosensing.^153^ More specifically, there have been many works on utilizing graphene and graphene oxide for their biosensing properties.^144,155–157^ For instance, Jin *et al.* demonstrated a functionalized graphene oxide wrapped around SiO_2_ that possessed superior RNA sensitivity and a limit of detection up to 1 fM, with the potential to show even higher sensitivity values.^158^ They showed that high electron conduction and larger surface area in spherical morphology are, in particular, effective at improving the sensitivity and limit of detection. However, as far as pathogen detection is concerned, very few works and reviews based on graphene exist so far to the best of our knowledge. Most are based on various designs, such as field-effect transistors (FETs) and electrochemical systems based on graphene.^143,149,152,159–161^ The working principle of graphene-based FET biosensing design as a nanoelectronic biosensor relies on charge detection for sensing in which an electrical signal is generated upon attachment of biomolecules to the surface of the sensor as a result of charge-density change.^149,162^ Although the FET-based graphene biosensor provides easier mass-scale production with satisfying sensitivity, its limited sensing capability along with being damaging to living cells make its application restricted compared to analogous optical ones.^46,148,163,164^ The superb optical properties of graphene, such as broadband and saturation absorption, and great affinity to adsorb biomolecules as a result of *π*–*π* stacking on its surface, make graphene a very promising candidate for development of any optical 2DM–based biosensor^165,166^ [Fig. 18(b)]. We briefly discuss the potential of graphene-based optical biosensors toward development of a fast, accurate, point-of-care pathogen detection system.

**FIG. 19.**
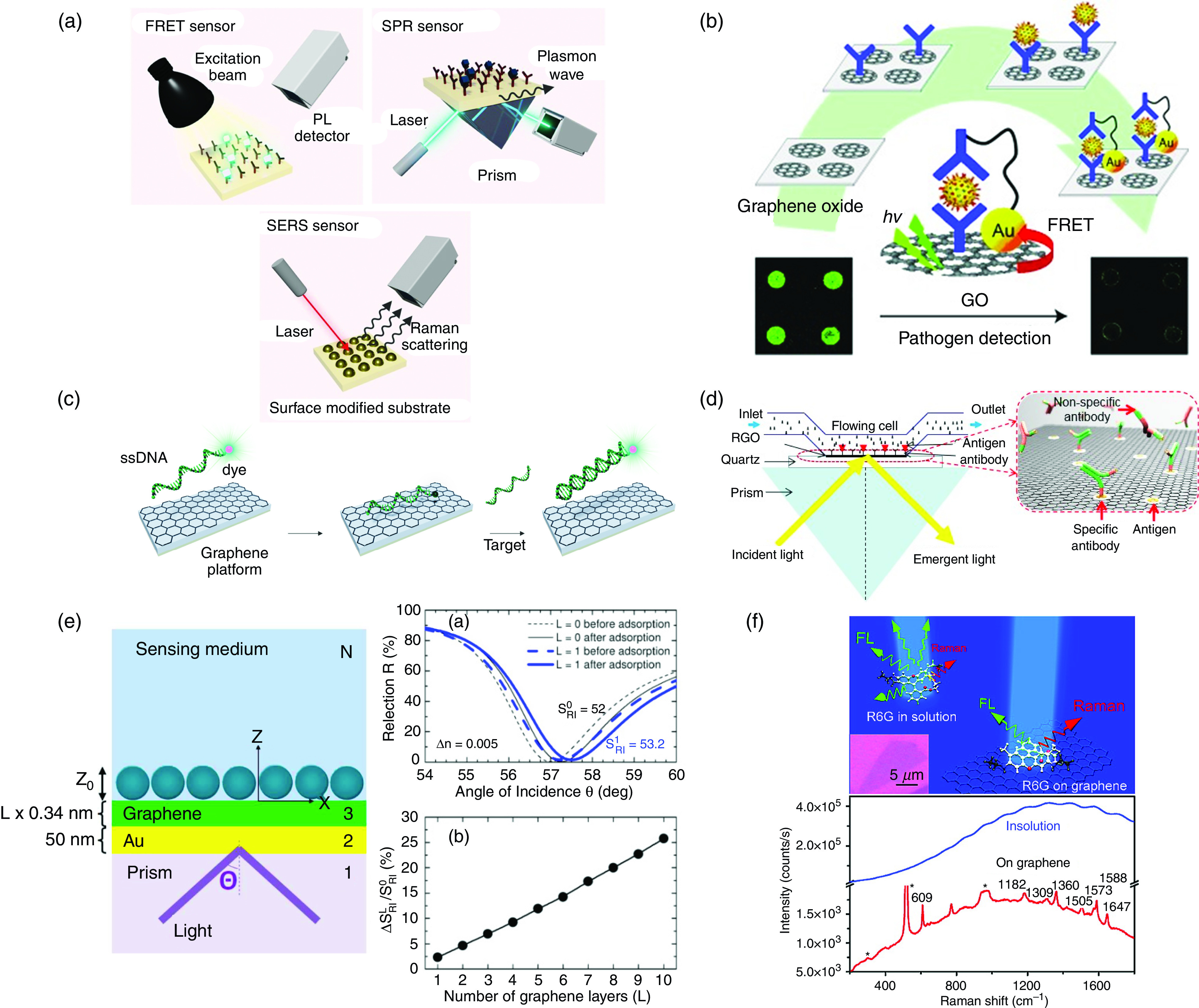
(a) Schematic representations of the working mechanisms of graphene-based optical biosensors. The three main methods discussed here are fluorescence, surface plasmon resonance (SPR), and surface-enhanced Raman spectroscopy (SERS).^40^ Reprinted with permission from Vermisoglou *et al.*, Biosens. Bioelectron. **166**, 112436 (2020). Copyright 2020 Elsevier.^40^ (b) Schematic representation of a graphene oxide (GO)–based immune biosensor. The antibodies for rotavirus are immobilized on the GO array, and the capture of target cells is observed through fluorescence quenching of GO by FRET between the GO and AuNPs. (c) Schematic showing the working mechanism of a graphene-based photoluminescence sensor utilizing GO as a fluorescence quenching platform for viral DNA detection.^171–173^ The fluorescent probes are quenched by GO upon attachment of fluorescent dye–conjugated nucleic acid onto their surface.^40^ (d) Schematic of an optical biosensor based on reduced graphene oxide (RGO). The inset demonstrates antigen–antibody binding to the RGO surface in which the dynamic can be measured in real time. (e) Left: Graphene-based SPR biosensor where L is the number of graphene layers and z is the thickness of biomolecule layer. Right: The effect of graphene and adsorption of biomolecules of the surface plasmon resonance curve and the sensitivity improvement upon adding a number of graphene layers. (f) Schematic illustration of the use of graphene to amplify the SERS signal. The photoluminescence suppression effect of graphene is shown with the Raman spectrum of R6G dye in water (blue line) vs in single-layer graphene (red line), showing a clear Raman signal without fluorescence background on graphene substrate compared to the solution. FL, fluorescence; *hv*, high voltage; PL, photoluminescence.

Optical biosensors based on 2DMs and, in particular, graphene can generally be divided into three main groups for sensing: fluorescence, SPR, and SERS [Fig. 19(a)]. Here, we briefly discuss each method and improvement of its sensing properties by utilizing graphene and graphene oxide.

### A. Fluorescence

The working mechanism of fluorescence biosensors is based on fluorescence quenching, fluorescence enhancement, or fluorescence resonance energy transfer (FRET).^149,167^ Graphene and its dioxides possess fluorescent quenching abilities that have enhanced visualization and detection of the target analyte through labeling biomolecules, such as ssDNA with fluorescent dye. The fluorescent-labeled DNA can be immobilized on the sensing area through *π*–*π* interaction between DNA probe and graphene surface.^149,168^ Jung *et al.*^99^ have demonstrated a GO-based immune biosensor to detect pathogens with high sensitivity and selectivity by GO photoluminescence quenching realized by FRET between AuNPs and GO [Fig. 19(b)]. Liu *et al.*^169^ and He *et al.*^170^ have also shown different complex sensing systems based on the high quenching ability and different affinity of GO toward DNA for complex sequence-specific DNA detection [Fig. 19(c)].

### B. SPR and LSPR

An SPR and LSPR biosensing platform conjugated with graphene has been developed to enhance the optical biosensor's sensitivity through amplifying detectable signal compared to conventional plasmonic materials.^149^ The conventional methods rely heavily on using biosensors based on gold and silver materials; however, the limitation of these metallic materials, like silver oxidization and poor adsorption of biomolecules on gold surface, have turned attention to utilizing a graphene-based optical SPR biosensor. The gold-coated graphene has a better biological sensing ability by adsorbing biomolecules better through *π*–*π* stacking. The effect of adding a graphene layer to the gold surface in a prism-based SPR has been shown by Wu *et al.*^171^ [Fig. 19(e)]. It has been demonstrated that the addition of graphene layer (L) would increase the sensitivity by $\left(1+0.025\;L\right)$ × *γ* (where *γ* > 1), where *γ* is the adsorption efficiency enhancement factor. It has been shown that graphene increases the sensitivity to refractive index change by 25% for L = 10 and, therefore, S increases five times in the case where graphene adsorbs four times more biomolecules (*γ* = 4).

### C. SERS

A Raman spectroscopy working mechanism is based on measuring inelastic scattering of light, which provides a footprint regarding the molecular or material structure. However, the Raman scattering from organic molecules is weak and needs to be amplified through applicable methods to be used as a biosensing mechanism. The enhancement of Raman signal can be accomplished by adding metal nanostructures due to the SPR effect. The graphene can be used as a very effective SERS active substrate as it can extinguish photoluminescence of fluorescent dyes and eliminate fluorescence background. Xie *et al.*^172^ have shown the photoluminescence suppression effect in a graphene substrate grown on a SiO_2_/Si surface due to electron transfer and energy transfer between the graphene and R6G dye molecules [Fig. 19(f)].

As mentioned, a wide range of graphene-based optical biosensors based on fluorescence, SERS, and SPR has been developed in recent years, among which few possess sufficient sensitivity and specificity to be utilized for SARS-CoV-2 detection (Table IV).^174–179^ Lee *et al.* showed a highly sensitive plasmonic/magnetic hybrid nanomaterial graphene-based platform possessing an attractive combination for virus detection: plasmonic and magnetic effects. They used a magnetic field for separation of target virus from impurities along with the plasmonic substrate for fluoroimmunosensing to detect influenza virus (H1N1) with LOD of 7.27 fg/ml.^180^ Graphene oxide was also used by Jeong *et al.* as a base for a facile fluorometric system for detection of influenza viral genes.^177^ Furthermore, an optical biosensor based on functionalized graphene oxide and cadmium sulfide quantum dots (CdS-NH_2_GO) has been developed to function through the surface plasmon resonance principle. The high binding affinity of targeted dengue virus envelope (DENV) E-proteins to the surface resulted in a high detection sensitivity of 0.08 pM. The low absorption of graphene and small photocurrent are a driving force to use graphene quantum dots (GQDs), which show a significant photoluminescence that can be improved by functionalization and doping of graphene.^40^ All the aforementioned methods show the potential of combining graphene-based materials with nanomaterials in COVID-19 optical biosensors. The final sensitivity can be improved by increasing the graphene-based sensing surface and proper surface functionalization with SARS-CoV-2 antibody or nucleic acid.

**TABLE IV.** Graphene- and graphene oxide–based optical biosensors with state-of-the-art limit of detection.^180^

Material	Detected virus	LOD	Ref.
GO	H1N1	3.8 pg/ml (0.2 pM)	177
RGO	Dengue	0.08 pM	178
RGO	Hepatitis C	10 fM	179
G/Au-FeONPs^a^	H1N1	7.27 fg/ml (0.11fM)	180

^a^ G/Au-FEONP, graphene/Au-FeO nanoparticle.

## VI. CONCLUSION

Emerging pandemic and epidemic diseases, like COVID-19, bring out a high demand for advancement and research in medical detection and treatment methods. The optical biosensors provide a fast detection (<1 min) of such a virus at very low concentrations (∼1 fM). However, they need to be designed and functionalized to be the most absorptive to the target analyte. The ideal label-free biosensor is cost-effective, disposable or reusable, compact, and semiautomatic. Although most efforts in biosensors have been focused on protein biomarkers, other targets, such as small molecules and nucleic acids, are crucial in expanding the application of biosensors, including optical ones. A common challenge for optical biosensors is to reach the capability of performing the measurement in real complex samples, avoiding, or limiting, the sample preparation phase. Developing a label-free biosensor is aligned with that purpose because the need for onsite detection techniques is increasing as the world after the COVID-19 pandemic will never be anything like before.

## AUTHORS' CONTRIBUTIONS

A.A., C.W., and K.M.Y. contributed equally to this work.

## Data Availability

The data that support the findings of this study are available from the corresponding author upon reasonable request.
